# Cost-effectiveness Analysis of Robotic Versus Open Pancreatoduodenectomy (DIPLOMA-2): A Multicenter International Randomized Trial

**DOI:** 10.1097/SLA.0000000000007188

**Published:** 2026-08-14

**Authors:** Julia E. Menso, Caro L. Bruna, Nine de Graaf, Anouk M.L.H. Emmen, Mahsoem Ali, Bergthor Björnsson, Ugo Boggi, Olivier R. Busch, Annalisa Comandatore, Freek Daams, Giovanni Ferrari, Sebastiaan Festen, Jony van Hilst, Ignace H.J.T. de Hingh, Mathieu D’Hondt, Benedetto Ielpo, Tobias Keck, Bas Groot Koerkamp, Daan J. Lips, Misha D.P. Luyer, J. Sven Mieog, Luca Morelli, I. Quintus Molenaar, Marco Ramera, Francesco Sucameli, Roeland F. de Wilde, Patrick Maisonneuve, Gudrun M.W. Björnelv, Mohammad Abu Hilal, Marc G. Besselink, Åsmund A. Fretland, for the European Consortium on Minimally Invasive Pancreatic Surgery (E-MIPS), Bert Bonsing

**Affiliations:** 1 Department of Surgery, Amsterdam UMC, location University of Amsterdam, Amsterdam, The Netherlands; 2 Cancer Center Amsterdam, The Netherlands; 3 Department of Surgery, OLVG, Amsterdam, The Netherlands; 4 Department of Surgery, Amsterdam UMC, location Vrije Universiteit, Amsterdam, The Netherlands; 5 Department of Biomedical and Clinical Sciences, Linköping University, Linköping, Sweden; 6 Clinical Department of Surgery in Linköping, Region Östergötland, Linköping, Sweden; 7 Department of Surgery, Universitá di Pisa, Pisa, Italy; 8 Department of Translational Research and New Technologies in Medicine and Surgery, General Surgery Unit, University of Pisa, Pisa, Italy; 9 Department of Surgery, Niguarda Ca’Granda Hospital, Milan, Italy; 10 Department of Surgery, Catharina Hospital, Eindhoven, The Netherlands; 11 Department of Digestive and Hepatobiliary/Pancreatic Surgery, Groeninge Hospital, Kortrijk, Belgium; 12 Department of Surgery, Hospital del Mar, Barcelona, Spain; 13 Department of Surgery, UKSH campus Lübeck, Lübeck, Germany; 14 Department of Surgery, Erasmus MC, Rotterdam, The Netherlands; 15 Department of Surgery, Medisch Spectrum Twente, Enschede, The Netherlands; 16 Department of Surgery, Leiden University Medical Center, Leiden, The Netherlands; 17 Department of Surgery, UMC Utrecht Cancer Center and St Antonius Hospital Nieuwegein: Regional Academic Cancer Center Utrecht, Utrecht, The Netherlands; 18 Department of General Surgery, Fondazione Poliambulanza Istituto Ospedaliero, Brescia, Italy; 19 Department of Clinical and Experimental Sciences, Università degli Studi di Brescia, Brescia, Italy; 20 Division of Epidemiology and Biostatistics, IEO European Institute of Oncology IRCCS, Milan, Italy; 21 Department of Health Management and Health Economics, Institute of Health and Society, University of Oslo, Oslo, Norway; 22 Department of Surgery, School of Medicine, University of Jordan, Amman 11942, Jordan; 23 Department of Surgery, University Hospital Southampton NHS Foundation Trust, Southampton, UK; 24 Department of Hepato-Pancreato-Biliary Surgery, and Institute of Clinical Medicine, University of Oslo, The Intervention Centre, Oslo University Hospital, Oslo, Norway

**Keywords:** cost-analysis, cost-effectiveness, economic evaluation, quality-adjusted life years, quality of life, robot-assisted pancreatoduodenectomy, pancreatic surgery

## Abstract

**Background:**

Although robot-assisted pancreatoduodenectomy (RPD) enhances patient recovery, it could be associated with higher costs than open pancreatoduodenectomy (OPD), potentially limiting its widespread adoption. High-level evidence comparing costs of RPD and OPD is lacking. This analysis in a randomized trial compared costs and cost-effectiveness between RPD and OPD.

**Methods:**

Predefined cost-effectiveness analysis in the international randomized DIPLOMA-2 trial (ISRCTN27483786) comparing RPD and OPD in patients with primary resectable neoplasm in 14 centers in 6 European countries (2022–2023). Primary outcomes were mean total hospital costs (euro) at 6 months postoperatively and cost-effectiveness based on costs per quality-adjusted life year (QALY; incremental cost-utility ratio). Three analyses were performed: (1) costs, (2) QALYs including Health-Related Quality of Life (HR-QoL), and (3) cost-effectiveness with probabilities at various willingness-to-pay (WTP) thresholds.

**Results:**

Overall, 268 patients were included (170 RPD, 98 OPD). No significant difference in total hospital costs was found between RPD and OPD (€30,956 vs. €28,271, *P*=0.538). The intraoperative costs were €5,491 higher with RPD (€11,906 vs. €6,451, *P*<0.001), whereas postoperative costs were €2806 lower (€19,051 vs. €21,856, *P*=0.518). The HR-QoL index scores were comparable at 1 and 3 months, but worse at 6 months with RPD (Δ−0.08 [−0.15 to −0.001]). Consequently, over 6 months a QALY loss was observed with RPD (Δ−0.02 [−0.07 to 0.01]) with a <30% probability of RPD being cost-effective across various WTP thresholds.

**Conclusion:**

This analysis in a randomized trial found no significant differences in costs between RPD and OPD up to 6 months. Nonsignificantly lower QALYs at 6 months with RPD resulted in a <30% probability that RPD is cost-effective compared with OPD within 6 months after surgery in selected patients in high-volume centers.

Pancreatoduodenectomy is among the most complex and high-risk surgical procedures, with major morbidity occurring in 25% of patients leading to prolonged time to recovery.^1,2^ Robot-assisted pancreatoduodenectomy (RPD) was introduced to reduce surgical trauma and enhance postoperative recovery. Four randomized controlled trials (RCTs) in selected patients and experienced centers have confirmed that RPD reduces blood loss, time to postoperative recovery, and hospital stay as compared with OPD.^3–5^

Robot-assisted procedures are expensive due to the required investment in the robotic system, related equipment, system maintenance, and prolonged operative time.^6^ These higher costs may hamper the widespread adoption of RPD. However, with growing experience, outcomes of RPD may improve, leading to reduced total hospital costs and ultimately comparable costs to OPD.^7,8^ Since postoperative pancreatic fistula (POPF) is the main driver of morbidity and thus hospital costs after pancreatoduodenectomy, its reduction may mitigate total hospital costs.^9–12^ According to the IDEAL framework for surgical innovation, comparative studies on robotic versus open surgery should evaluate cost-effectiveness and quality of life outcomes in addition to clinical outcome.^13^ A recent meta-analysis showed that the methodology of cost analyses in RCTs is typically suboptimal as analyses often do not take all surgical costs, for example, robot purchase/maintenance costs into account.^14^ To date, prospective studies focusing on costs comparing RPD and OPD, including the assessment of Health-Related Quality of Life (HR-QoL) are lacking.

This predefined subanalysis within the DIPLOMA-2 international randomized trial compared total hospital costs and cost-effectiveness between RPD and OPD, evaluating both direct and indirect health care expenditures, including robot purchase/maintenance costs and HR-QoL up to 6 months postoperatively.

## METHODS

### DIPLOMA-2 Trial

This study was a predefined analysis within the international randomized controlled patient-blinded noninferiority DIPLOMA-2 trial which compared (2:1 randomization) minimally invasive (MIPD; ie, robot-assisted and laparoscopic) and open pancreatoduodenectomy (OPD) in patients with primary resectable pancreatic and periampullary lesions in 14 high-volume centers across 6 European countries between January 2022 and September 2023.^5^ For the present analysis, the 20 patients undergoing laparoscopic pancreatoduodenectomy were excluded and follow-up of all patients was extended to 6 months postoperatively. Ethical approval was granted by the Medical Ethical Committee of Amsterdam UMC in January 2022. This study was reported according to the Consolidated Health Economic Evaluation Reporting Standards (CHEERS 2022) guideline (Supplementary Table 1, Supplemental Digital Content 1, https://links.lww.com/SLA/F927).^15^ Details on patient selection, data collection, clinical outcome, and health care use are reported in Supplementary file 1, Supplemental Digital Content 1, https://links.lww.com/SLA/F927.

### Outcomes

The primary outcomes were total hospital costs per patient (euro) and cost-effectiveness estimated as costs per quality-adjusted life years (QALYs). Secondary outcomes were major complications (Clavien-Dindo grade ≥3), length of primary stay (days), total length of stay (including readmissions, days), readmission, reinterventions, MC/IC admission, mortality, and HR-QoL. The follow-up was up to 6 months postoperatively or in-hospital if (re)admission exceeded 6 months after surgery.

### Hospital Costs

Costs per patient were calculated using a bottom-up approach by multiplying the individual health care resource use by unit costs. Unit costs were estimated based on the Dutch manual for costing in health care research.^16^ The index year for costs was 2023 and yearly consumer price indices were used to standardize unit costs assessed in different calendar years as reported by Statistics Netherlands (CBS; Supplementary Table 2, Supplemental Digital Content 1, https://links.lww.com/SLA/F927).^17^ Details on costs (eg, robot purchase/maintenance costs) are reported in Supplementary file 1 and details on unit costs in Supplementary Table 2, Supplemental Digital Content 1, https://links.lww.com/SLA/F927

### Cost-effectiveness

Cost-effectiveness was presented as the mean incremental cost-effectiveness ratio (ICER; Δcosts/ΔQALYs = [cost OPD – cost RPD]/[QALYs OPD – QALYs RPD], ie, difference in mean total hospital costs divided by the difference in mean QALYs with RPD vs. OPD).^18^ Details on QALYs (including HR-QoL) are presented in Supplementary file 1, Supplemental Digital Content 1, https://links.lww.com/SLA/F927. A positive ICER indicates that RPD is more effective and more costly or less effective and less costly than OPD, whereas a negative ICER indicates that RPD is more effective and less costly (ie, always cost-effective, RPD dominates OPD) or less effective and more costly (ie, never cost-effective, RPD is dominated by OPD). If the ICER falls below a predefined WTP threshold, RPD is considered cost-effective. Because the accepted value for one QALY varies per country, this study used the Dutch WTP threshold of €80,000.^19^

### Statistical Analysis

Details on the statistical analyses are reported in Supplementary file 1, Supplemental Digital Content 1, https://links.lww.com/SLA/F927.

## RESULTS

### Participants

Overall, 288 patients were randomized (190 MIPD, 98 OPD). In the MIPD group, 170 patients (89.5%) received RPD and were included in this analysis. One patient in the RPD group and one patient in the OPD group did not undergo resection due to intraoperatively diagnosed metastasized or locally advanced disease and were not included in this analysis (Supplementary Fig. 1, Supplemental Digital Content 1, https://links.lww.com/SLA/F927). Baseline characteristics were comparable between the groups (Supplementary Table 8, Supplemental Digital Content 2, https://links.lww.com/SLA/F968).

### Intraoperative and Histopathologic Outcomes

In the RPD group, 8.2% of patients underwent conversion from RPD to OPD. The RPD group had a longer median operative time and less median blood loss. The rates of patients with a small main pancreatic duct (≤3 mm), soft pancreas, vascular resection, and additional organ resection were comparable between groups, as was the incidence of final diagnosis PDAC and any type of cancer. Among 78 patients with final diagnosis PDAC, the R0-resection rate was nonsignificantly higher in the RPD group (Supplementary Table 3, Supplemental Digital Content 1, https://links.lww.com/SLA/F927).

### Postoperative Outcome

No difference in the major complication rate up to 6 months postoperatively was observed after RPD and OPD. The rate of POPF grade B/C was lower after RPD, whereas the rates of PPH grade B/C, DGE grade B/C, and bile leak grade B/C were comparable. A nonsignificant difference was observed in 6-month overall mortality (9.4% vs. 5.1%, *P*=0.206) and complication-related mortality (4.1% vs. 2.0%, *P*=0.363) between RPD and OPD. The median time to functional recovery was 1 day shorter after RPD and the median length of primary stay 3 days shorter. The rates of MC/IC admission, readmission, unplanned hospital visits, endoscopic-, radiological-, and surgical reinterventions were comparable. The receipt of adjuvant therapy for PDAC and any type of cancer were also comparable (Table 1).

**TABLE 1:** Postoperative Outcome in Patients Undergoing RPD Versus OPD in the DIPLOMA-2 Randomized Trial Up To 6 Months

Postoperative Complications	RPD (n=170) (%)	OPD (n=98) (%)	*P*
Major complications (CD ≥3)	96 (56.5)	54 (55.1)	0.828
POPF grade B/C	40 (23.5)	35 (35.7)	**0.032**
PPH grade B/C	14 (8.2)	10 (10.2)	0.587
DGE grade B/C	34 (20.0)	26 (26.5)	0.217
Bile leak grade B/C	13 (7.6)	6 (6.1)	0.640
Wound infection (CDC)	25 (14.7)	23 (23.5)	0.072
Superficial	10 (5.9)	13 (13.3)	0.196^
Deep	5 (2.9)	3 (3.1)	
Intra-abdominal collection	10 (5.9)	7 (7.1)	
Organ failure	14 (8.2)	5 (5.1)	0.336
In-hospital/6-month mortality	16 (9.4)	5 (5.1)	0.206
Surgery complication-related	7 (4.1)	2 (2.0)	0.363
Disease progression-related	8 (4.7)	2 (2.0)	0.268
*Missing*	*0*	*1*	
Health care use			
Time to functional recovery (days)	7 (5–11)	8 (6–14)	**0.042**
Length of primary stay	9 (6–15)	12 (7–20)	**0.039**
Total length of stay*	13 (8–22)	15 (8–27)	0.308
≤6 d	26 (15.3%)	11 (11.2%)	0.352
Readmission	64 (37.6%)	32 (32.7%)	0.412
MC/IC admission	22 (12.9%)	15 (15.3%)	0.589
Unplanned hospital visits†	37 (21.8)	24 (24.5)	0.608
Endoscopic intervention	49 (28.8)	25 (25.5)	0.559
Radiologic intervention	69 (40.6)	42 (42.9)	0.716
Surgical reintervention	21 (12.4)	8 (8.2)	0.288
Blood transfusion	31 (18.2)	19 (19.4)	0.816
Adjuvant therapy for PDAC	45 (88.2)	23 (88.5)	0.977
*Missing*	*1*	*0*	
Adjuvant therapy for any type of cancer	63 (48.8)	33 (50.8)	0.799
*Missing*	*2*	*1*	

Legend ‘ementary table 4: Values are presented in medians with interquartile ranges and frequencies with percentages. CD indicates Clavien-Dindo classification; CDC, Centers for Disease Control and Prevention; DGE, delayed gastric emptying; IC, intensive care unit; MC, medium care unit; OPD, open pancreatoduodenectomy; PDAC, pancreatic ductal adenocarcinoma; POPF, postoperative pancreatic fistula; PPH, postpancreatectomy hemorrhage; RPD, robot-assisted pancreatoduodenectomy.

* Total length of stay includes readmission(s).

† Emergency room and unplanned outpatient clinic visits.

^ *P*-value calculated over the group variable.

### Hospital Costs

No statistically significant difference in mean total hospital costs was found between RPD and OPD (€30,956 vs. €28,271, *P*=0.538). The mean intraoperative costs, including fixed and additional costs, were €5,491 higher in RPD (€11,906 vs. €6,451, *P*<0.001), whereas the mean postoperative costs were nonsignificantly €2806 lower for RPD (€19,051 vs. €21,856, *P*=0.518).

When excluding robot purchase/maintenance costs, the difference in total hospital costs decreased. In 195 patients with preoperative non-PDAC (121 RPD, 74 OPD), total hospital costs remained nonsignificantly higher for RPD. In 71 patients with preoperative PDAC (49 RPD, 22 OPD), total hospital costs were nonsignificantly lower for RPD. In 170 patients with a high-risk for POPF (110 RPD, 60 OPD) and in 98 low-risk patients (60 RPD, 38 OPD), total hospital costs were comparable (Supplementary Table 8, Supplemental Digital Content 3, https://links.lww.com/SLA/F969).

### Health-related Quality of Life and Quality-adjusted Life Years

In the RPD group, adjusted HR-QoL index scores were 0.84 (95% CI: 0.82–0.86) at baseline and decreased to 0.74 (0.69–0.78) at 1 month, 0.77 (0.73–0.82) at 3 months and 0.72 (0.66–0.77) at 6 months after surgery. In the OPD group, HR-QoL index scores were 0.80 (0.77–0.84) at baseline and decreased to 0.73 (0.68–0.77) at 1 month before stabilizing to baseline value with 0.81 (0.77–0.84) at 3 months and 0.79 (0.74 to 0.84) at 6 months. Adjusted HR-QoL index scores were comparable between RPD and OPD at 1 month (Δ0.01 [−0.05 to 0.07]) and 3 months after surgery (Δ−0.03 [−0.09–0.03]), but worse at 6 months with RPD (Δ−0.08 [−0.15 to −0.001]; Fig. 1). Consequently, patients undergoing RPD had nonsignificantly lower QALYs (Δ−0.01 [−0.06 to 0.03] and −0.02 [−0.07 to 0.01] without and with covariate adjustment, respectively).

**FIGURE 1:**
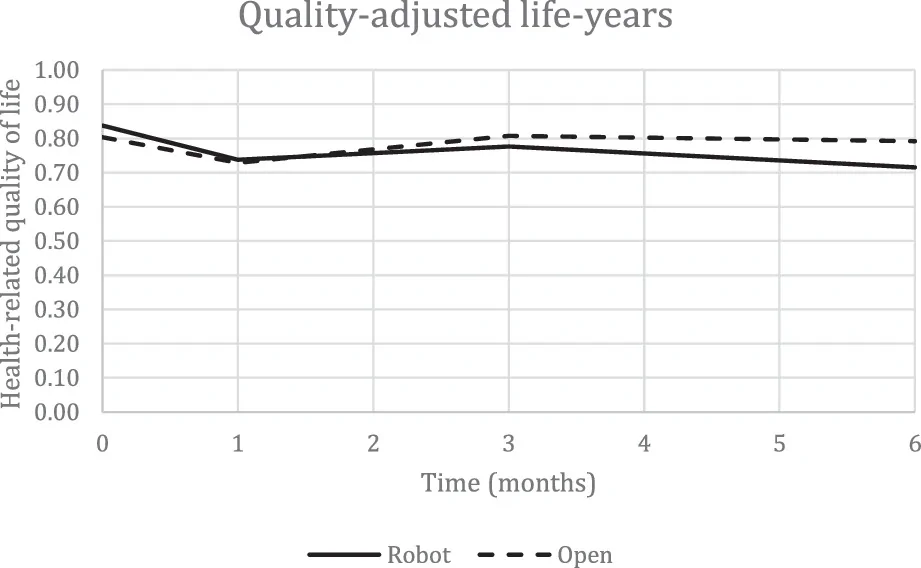
Health-related quality of life (EQ-5D-5L) over time in all patients undergoing RPD versus OPD in the DIPLOMA-2 randomized trial up to 6 months. Health-related quality of life (HR-QoL; Y-axis, utility) over time (X-axis, months), based on the EuroQol Five Dimensions Five-Level (EQ-5D-5L) questionnaire, adjusted for HR-QoL at baseline. Quality-adjusted life years (QALYs) were estimated as HR-QoL times time (ie, area under the curve).

When including only survivors (n=247), HR-QoL index scores at 6 months were comparable between RPD and OPD (0.81 vs. 0.84; Δ−0.04 [−0.09 to 0.02]; Fig. 2). When including survivors and complication-related deaths only (n=256), HR-QoL index scores at 6 months also became comparable (0.77 vs. 0.82, Δ−0.05 [−0.11 to 0.01]; Supplementary Fig. 2, Supplemental Digital Content 1, https://links.lww.com/SLA/F927). Details on the handling of missingness and the other sensitivity analyses (EQ-5D-5L index score) are reported in Supplementary file 2 and Supplementary Figures 3–6, Supplemental Digital Content 1, https://links.lww.com/SLA/F927. Details on HR-QoL per domain (EQ-5D-5L, QLQ-C30 and QLQ-PAN26 descriptive values) are presented in Supplementary Tables 4–7, Supplemental Digital Content 1, https://links.lww.com/SLA/F927.

**FIGURE 2:**
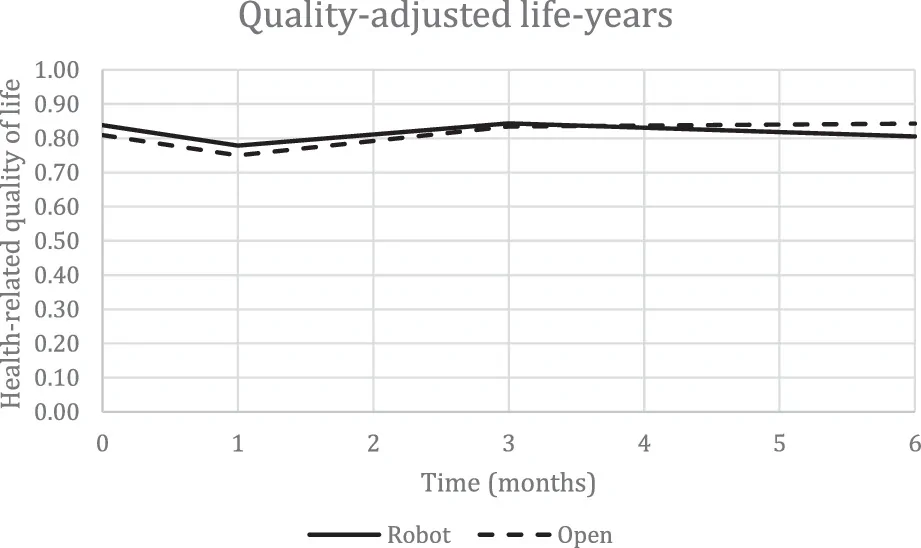
Health-related quality of life (EQ-5D-5L) over time in patients undergoing RPD versus OPD in the DIPLOMA-2 randomized trial up to 6 months in survivors only. Health-Related Quality of Life (HR-QoL; Y-axis, utility) over time (X-axis, months) in survivors only (n=247, ie, excluding 21 deceased patients), based on the EuroQol Five Dimensions Five-Level (EQ-5D-5L) questionnaire, adjusted for HR-QoL at baseline. Quality-adjusted life years (QALYs) were estimated as HR-QoL times time (ie, area under the curve).

### Cost-effectiveness

Robot-assisted pancreatoduodenectomy was less likely to be cost-effective compared with OPD, because of nonsignificantly higher total hospital costs and QALY loss compared with OPD. The cost-effectiveness acceptability curve demonstrated a low probability of RPD being cost-effective for all WTP thresholds (Supplementary Figure 13, Supplemental Digital Content 4, https://links.lww.com/SLA/F970). At the Dutch WTP threshold of €80.000 per QALY, the probabilities of being cost-effective were 18% for RPD and 82% for OPD.

When including survivors only (n=247), RPD was still dominated by OPD (QALYs Δ−0.003, incremental costs €2,858 per QALY, 24% probability of RPD being cost-effective at the Dutch WTP threshold of €80.000; Supplementary Fig. 7, Supplemental Digital Content 1, https://links.lww.com/SLA/F927). When including survivors and complication-related deaths only (n=256), RPD was also still dominated by OPD (QALYs Δ−0.014, incremental costs €2,662 per QALY, 21% probability of RPD being cost-effective; Supplementary Fig. 8, Supplemental Digital Content 1, https://links.lww.com/SLA/F927). Details on the other sensitivity analyses are reported in Supplementary file 2 and Supplementary Figures 9–12, Supplemental Digital Content 1, https://links.lww.com/SLA/F927.

## DISCUSSION

This first short-term cost-effectiveness analysis in a randomized trial comparing RPD versus OPD found no statistically significant difference in mean total hospital costs (RPD €30,956 vs. OPD €28,271). The HR-QoL was comparable at 1 month and 3 months after surgery, but worse at 6 months with RPD, resulting in nonsignificantly lower QALYs and a probability below 30% of RPD being cost-effective compared with OPD at any WTP threshold in selected patients in high-volume centers.

These results cannot be compared with earlier randomized studies. The higher intraoperative costs for RPD were partly offset by lower postoperative costs. Consequently, the mean total hospital costs were nonsignificantly €2,685 higher with RPD compared with OPD. These higher intraoperative costs are consistent with previous literature and largely attributable to the costs for purchase of the robotic system, maintenance, and instruments.^8,20^ The lower postoperative costs with RPD were largely driven by length of hospital stay, MC/IC admission, and surgical and vascular reinterventions, as previously reported.^12^ Although the primary hospital stay in this study was shorter after RPD than OPD, this initial advantage diminished due to nonsignificantly more readmissions after RPD, resulting in a comparable total length of hospital stay between groups. Two recent retrospective cost analyses from single high-volume centers in the Netherlands and Japan, including 588 and 120 pancreatoduodenectomies, respectively, also reported that the higher intraoperative costs with RPD were offset by shorter length of stay compared with OPD.^7,21^ These findings contrast with a meta-analysis, including 83,121 pancreatoduodenectomies from 16 cohort and case-control studies, which reported significantly higher total costs for RPD compared with OPD, despite the shorter primary length of stay after RPD.^22^

As total hospital costs for RPD and OPD may differ between subgroups, multiple sensitivity analyses were performed, with broadly unchanged results. In patients with PDAC only, RPD was nonsignificantly less costly than OPD (€27,544 vs. €31,852), whereas in patients with non-PDAC–with a higher fistula risk – total hospital costs were nonsignificantly higher for RPD (€32338 vs. €27,630). Although nonsignificant, these findings suggest that RPD in patients with PDAC (ie, a lower fistula risk) may have the highest chance to offset the increased intraoperative costs of RPD by reducing postoperative costs. Finally, opportunity costs warrant consideration. In the Netherlands–and likely in most European institutions–hospital reimbursement is tied to the entire treatment pathway rather than a fixed per-procedure payment regardless of the postoperative course in which complications may limit the ability to perform other billable procedures. As such, complications primarily increase costs through prolonged hospital stay and readmissions rather than lost revenue from missed procedures, as may occur in fee-for-service systems such as the United States. While shorter hospital stay may theoretically increase treatment capacity, these opportunity costs are indirect and difficult to quantify. Therefore, length of hospital stay was considered an appropriate surrogate measure in this context.

This study provides the first analysis of HR-QoL in a randomized trial comparing RPD versus OPD. Adjusted HR-QoL index scores were comparable between groups at 1 and 3 months after surgery. However, at 6 months postoperatively, the adjusted HR-QoL index score was worse with RPD across multiple analytic imputation strategies and sensitivity analyses. The adjusted HR-QoL descriptive values were also significantly worse after RPD across several EQ-5D-5L domains with consistent nonsignificant worse trends in the other domains, showing that this pattern was not solely driven by a single domain. In contrast, a meta-analysis, including 3244 pancreatic resections for pancreatic cancer from 22 studies, reported a decrease in HR-QoL until 3 months after surgery, whereafter this recovered to baseline level at 6 months postoperatively.^23^ The impaired HR-QoL after RPD versus OPD may be partly explained by the nonsignificantly higher 6-month overall mortality (9.4% vs. 5.1%) and non–complication-related mortality rates after RPD (4.1% vs. 2.0%), since deceased patients contributed a health utility index score of zero. Although less POPF grade B/C occurred after RPD compared with OPD, the impact of POPF may have been greater after RPD, as previously suggested in the literature.^2^ Importantly, in the sensitivity analyses in survivors only and excluding patients with non–complication-related mortality, the difference in adjusted HR-QoL at 6 months after RPD versus OPD attenuated, indicating that the observed difference is partly attributable to the nonsignificantly higher mortality rate rather than impairment of recovery-related HR-QoL among surviving patients. These findings highlight the fact that centers should monitor their mortality rates following RPD. Interestingly, in the 3 other RCTs (EUROPA, Beijing, PORTAL) the mortality rates of RPD were 0–1% although none of these studies reported outcomes until 6 months, including rates of cancer recurrence and HR-QoL.^3,4,24^ The HR-QoL may also be impacted by cancer recurrence, which is related to receipt of adjuvant therapy. In this study, no statistically significant difference was found between groups regarding final diagnosis PDAC (30.8% vs. 26.8%) or any type of cancer (77.5% vs. 68.8%), nor adjuvant therapy for PDAC (88.2% vs. 88.5%) or any type of cancer (48.8% vs. 50.8%). Therefore, these factors are unlikely to have influenced differences in HR-QoL.

The nonsignificantly higher total hospital costs and loss in QALYs resulted in ICER dominated by OPD, indicating that the probability of RPD being cost-effective is below 30% at any WTP threshold. The probability of RPD being cost-effective remained low in the sensitivity analyses excluding deceased patients–although the negative incremental QALY diminished–and excluding non–complication-related deaths, suggesting that the dominated ICER is not solely driven by the difference in mortality rate. Although less POPF was observed after RPD, the probability of RPD being cost-effective remained below 30% for both high- and low-risk patients. Although the probability of RPD being cost-effective was 34% in high-volume centers compared with 13% in low-volume centers, RPD was still less likely to be cost-effective when compared with OPD. Hence, this study could not identify patient subgroups in which RPD was cost-effective. Previous studies showed that RPD is more expensive in the early learning curve, emphasizing the impact and importance of annual volume and surgical experience.^21^ A recent meta-analysis on cost-effectiveness of MIPD versus OPD also reported that RPD was not cost-effective when compared with OPD. It is important to note that this meta-analysis only included one RCT on RPD versus OPD, that is, the single-center EUROPA trial from Heidelberg, comparing 29 patients undergoing RPD versus 33 OPD.^3,25^

The results of this study should be interpreted considering several limitations. First, because center-specific cost data were not publicly available, all hospital unit costs were calculated based on the prices of one center. Reimbursement structures vary across countries, with greater heterogeneity in market-driven health care systems, such as the United States, limiting direct the generalizability of the present findings beyond European centers. This study was conducted according to the Dutch financial system with episode-based payments (ie, largely fixed reimbursement per care episode), unlike fee-for-service models where cost differences may be more pronounced. Although absolute costs depend on reimbursement system, the incremental cost differences are likely largely comparable due to randomization. Second, the per-procedure costs for RPD in lease/purchase-based financial models are highly subjective to the center’s total volume of robotic surgical procedures, limiting comparability between centers. This study used a procedural cost of €2,150 per RPD, based on the annual volume and lease costs. To improve the generalizability of the results, potential intercountry variation was addressed in the sensitivity analysis excluding the robot purchase/maintenance costs, which still showed no statistically significant difference in total hospital costs between RPD and OPD, but decreased this difference considerably. In addition, purchase models may differ between countries and even institutions. The Average Manufacturer Price (AMP) model of Intuitive Surgical (Sunnyvale, CA) includes a fixed per-procedure price, including maintenance, which is lower than the procedural costs in this study. The per-procedure costs for OPD are slightly underestimated, as costs of diagnostic laparoscopy for OPD in patients with PDAC were not taken into account. Third, no data were available on total time in the OR complex (patient arrival to exit) and postoperative diagnostic imaging, but the underestimation of hospital costs was minimized by including microcosts of surgical instruments and postoperative reinterventions. Fourth, this study included only in-hospital costs up to 6 months postoperatively. Costs of home care (eg, treatment for wound infection, which is more common after OPD^5^) were not collected. Fifth, the EQ-5D-5L index score was based on the preferences of the Dutch population, hence the individual HR-QoL scores are not directly translatable to other countries. Sixth, other factors–besides mortality and costs–may also contribute to uncertainty of the present findings (including the expected value of perfect information) and should be addressed in future studies. The major strength of this study is that this is the first prospective cost-effectiveness analysis of RPD versus OPD in an international randomized trial.

To conclude, this first short-term cost analysis in a randomized trial comparing RPD and OPD found that there is a probability below 30% that RPD is cost-effective compared with OPD within 6 months postoperatively at any WTP threshold in selected patients in high-volume centers. Although total hospital costs and HR-QoL up to 3 months after surgery were comparable, HR-QoL at 6 months declined after RPD, resulting in a QALY loss and higher incremental costs compared with OPD. These findings underscore the importance of low procedural mortality after RPD, which should be monitored in registries, and the need for careful, comprehensive evaluation of HR-QoL and long-term cost-effectiveness of RPD (eg, the ongoing DIPLOMA-2×2 trial).

## Supplementary Material

## DISCUSSANTS

### Massimo Falconi (Milano, Italy)

Thank you, Dr. Menso. Congratulations on the very interesting study and the great methodology you have applied to it. The first question concerns how you can interpret the decreased quality of life after robotic procedures compared with the open procedure, and whether this represents a true patient-reported signal, or mainly a consequence of the way mortality was incorporated into the study analysis. More broadly, how would you eventually redesign potential quality metrics in a future study?

My second question concerns the usual challenge of cost analyses. How can findings from a cost analysis performed, for example, in the Netherlands be translated to other health care systems, where you have completely different insurance reimbursement models and hospital compensation for the procedure may differ substantially?

Finally, have you considered the specific metrics for a subgroup analysis, such as in patients with pancreatic ductal adenocarcinoma, those at high risk for fistula, or procedures performed in high-volume robotic centers?

### Response From Julia E. Menso (Amsterdam, The Netherlands)

Firstly, regarding quality of life, this is partly explained by the nonsignificantly higher mortality rates, because deceased patients were appointed an EQ. 5D index score of 0. Indeed, the sensitivity analysis showed that this difference in quality of life was attenuated and was no longer significant. Yet, this isn’t solely based on mortality, as the probability of robotic Whipple being cost-effective remains low. In a separate analysis focusing only on quality-of-life descriptive values, similar trends were observed per domain of the QLQ-C30 and QLQ-PAN26 questionnaires. An explanation for the latter remains unclear. To conclude, the differences in quality of life are partly explained by the nonsignificantly higher mortality rates after robotic Whipple.

Regarding your second question, good outcome measurements should be established prior to the robotic Whipple, for example, by participating in a robotic Whipple training program, such as the LEARNBOT program. Appropriate outcome measurements for quality evaluation would be conversion, major morbidity, mortality, and failure to rescue. Regarding your other question on reimbursement models for the fee-for-service systems, it’s difficult to appoint specific prices and compare them, because they significantly vary between centers. This also depends on a center’s volume. Therefore, it’s highly recommended to perform robotic Whipple only in high-volume centers. Firstly, the sensitivity analysis stratified by annual volume showed that the probability of robotic Whipple being cost-effective was 34% in high-volume centers, compared with only 14% in low-volume centers. Besides, we published an Amsterdam UMC series in *Annals of Surgery* last year, comparing the cost of robotic versus open Whipple. We also compared the first 100 robotic Whipple patients with the second 100 robotic Whipple patients, showing that costs decreased by more than €11,000 per patient. It’s also important to note that, in the DIPLOMA-2 trial, the minimum required experience for surgeon participation was 60 robotic Whipples, not 100. There were centers that had not yet reached 100 procedures. Regarding your second question about subgroups, we recently performed an IPDMA, including all the individual patient data from the now 4 completed robot Whipple RCTs, to identify which patient subgroups might benefit the most. There was no specific patient subgroup that benefited more from either approach.

### Nicolò De Manzini (Trieste, Italy)

Compliments for your topics. Going beyond cost and cost-benefit, as you have shown us, we also have to consider sustainability in a world where health care money is limited. Have you calculated the CO2 footprint of robotic surgery, even in cases where the cost analysis shows a good result?

### Response From Julia E. Menso (Amsterdam, The Netherlands)

Thank you for your question. We aim to investigate this, but it remains very difficult to quantify these outcomes, so we’re not there yet. The ongoing DIPLOMA-3 trial, which compares robot versus laparoscopic left pancreatectomy, aims to identify benchmarks and values that may also be applicable to the Whipple procedure. However, that is to be continued.

### Safi Dokmak (Clichy, France)

Congratulations on this study. My question regards quality of life. Did you have the baseline quality of life of these patients? The robotic approach is performed in selected patients, some of whom have incidentally discovered disease, such as IPMN or neuroendocrine tumors. Probably, the baseline quality of life of these patients may be better than that of patients operated using the open approach, which could explain the difference in quality of life.

### Response From Julia E. Menso (Amsterdam, The Netherlands)

Baseline quality of life was better in the robotic group, but we performed the Manca correction to adjust for this difference. Even for the uncorrected analysis with a better baseline quality of life in the robotic group, robotic Whipple still showed worse outcomes, showing that the decrease was greater in the robotic group. Additional analyses investigated whether this difference could be attributed to potential differences in disease progression, but there was no statistically significant difference in oncological diagnosis or the receipt of adjuvant therapy.

### Dieter Hahnloser (Lausanne, Switzerland)

If I recall well, two-thirds of the costs were due to the operation itself, and one-third was due to what happened afterwards. The patient was blinded, but obviously, the surgeon wasn’t. How did the surgeon’s decision to prescribe more pain medication, change something, or keep the patient longer in the ICU influence costs? The surgeon knew it was an open procedure, and perhaps, prescribed more pain medication as a consequence. How do you think the surgeon’s decision could have affected both the patient outcomes and the overall costs?

### Response From Julia E. Menso (Amsterdam, The Netherlands)

Thank you. The trial included both open Whipple surgeons and robotic Whipple surgeons. However, the DIPLOMA-2 trial was designed as a pragmatic trial, reflecting real-world practice. If robotic Whipple surgeons tend to discharge patients earlier, then that does reflect real-life practice.

### Christiane Bruns (Cologne, Germany)

My question also pertains to quality of life. You showed that patients who underwent open procedures had worse quality of life during the first 6 months. However, you also showed that a Clavien-Dindo of more than 3 was comparable in both groups. Therefore, I then think that interventions, such as anastomotic insufficiency or bile leaks, should also have been comparable. How, then, do you explain the poorer quality of life reported by patients undergoing the open procedure compared with those undergoing the robotic approach? Do you think this is mainly a psychological effect, or could it be explained by patients undergoing open procedures remaining in the hospital longer after these types of interventions than those undergoing robotic procedures?

### Response From Julia E. Menso (Amsterdam, The Netherlands)

Thank you. There was no statistically significant difference in the quality of life at 1 and 3 months after surgery, but the rate of postoperative pancreatic fistula grade B/C was lower after robotic Whipple, which could explain the nonsignificant difference in quality of life during the first three months.
