# Supplementary material for: Cost-effectiveness Analysis of Robotic Versus Open Pancreatoduodenectomy (DIPLOMA-2): A Multicenter International Randomized Trial
Source: Ann Surg. 2026 Aug 14;284(5):920–7. doi: 10.1097/SLA.0000000000007188 (PMC13652126; doi:10.1097/SLA.0000000000007188)
Supplement: Supplementary file 1 [file sla-284-0920-s001.docx]

**Cost-effectiveness Analysis of Robotic versus Open Pancreato-duodenectomy (DIPLOMA-2): A Multicenter International Randomized Trial**

Menso, Julia E, MD*^1,2^, Bruna, Caro L, MD*^1,2^, de Graaf, Nine, MD PhD^1,2,3^, Emmen, Anouk MLH, MD PhD^1,2^, Mahsoem Ali, BSc^2,4^, Björnsson, Bergthor, MD PhD^5,6^, Boggi, Ugo, MD PhD^7^, Busch, Olivier R, MD PhD^1,2^, Comandatore, Annalisa, MD^8^, Daams, Freek, MD PhD^2,4^, Ferrari, Giovanni, MD^9^, Festen, Sebastiaan, MD PhD^3^, van Hilst, Jony, MD MSc PhD^1,2,3^, de Hingh, Ignace HJT, MD PhD^10^, D’Hondt, Mathieu, MD PhD^11^, Ielpo, Benedetto, MD PhD^12^, Keck, Tobias, MD PhD^13^, Groot Koerkamp, Bas, MD MSc PhD^14^, Lips, Daan J, MD PhD^15^, Luyer, Misha DP, MD PhD^10^, Mieog, J Sven D, MD PhD^16^, Morelli, Luca, MD PhD^8^, Molenaar, I Quintus, MD PhD^17^, Ramera, Marco, MD^18,19^, Sucameli, Francesco, MD^18^, de Wilde, Roeland F, MD PhD^14^, Maisonneuve, Patrick, Dipl.Eng^20^, Björnelv, Gudrun MW, MSc PhD^21^*, Abu Hilal, Mohammad, MD PhD^22,23^*, Besselink, Marc G, MD MSc PhD^1,2^*, Fretland, Åsmund A, MD PhD^24^* for the European Consortium on Minimally Invasive Pancreatic Surgery (E-MIPS)

*Shared first/senior authorship

**Collaborators:** Bert Bonsing^16^, Michael Ginesini^7^, Celine De Meyere^11^, Joost Nederend^10^, Per Sandström^5,6^, Joost Sprakel^15^, Ulrich Wellner^13^

**Affiliations**

1 Amsterdam UMC, location University of Amsterdam, Department of Surgery, Amsterdam, The Netherlands

2 Cancer Center Amsterdam, The Netherlands

3 OLVG, Department of Surgery, Amsterdam, The Netherlands

4 Amsterdam UMC, location Vrije Universiteit, Department of Surgery, Amsterdam, The Netherlands

5 Department of Biomedical and Clinical Sciences, Linköping University, Linköping, Sweden

6 Clinical Department of Surgery in Linköping, Region Östergötland, Linköping, Sweden

7 Universitá di Pisa, Department of Surgery, Pisa, Italy

8 University of Pisa, General Surgery Unit, Department of Translational Research and New Technologies in Medicine and Surgery, Pisa, Italy

9 Niguarda Ca’Granda Hospital, Department of Surgery, Milan, Italy

10 Catharina Hospital, Department of Surgery, Eindhoven, The Netherlands

11 Groeninge Hospital, Department of Digestive and Hepatobiliary/Pancreatic Surgery, Kortrijk, Belgium

12 Hospital del Mar, Department of Surgery, Barcelona, Spain

13 UKSH campus Lübeck, Department of Surgery, Lübeck, Germany

14 Erasmus MC, Department of Surgery, Rotterdam, The Netherlands

15 Medisch Spectrum Twente, Department of Surgery, Enschede, The Netherlands

16 Leiden University Medical Center, Department of Surgery, Leiden, The Netherlands

17 Department of Surgery, UMC Utrecht Cancer Center and St Antonius Hospital Nieuwegein: Regional Academic Cancer Center Utrecht, Utrecht, The Netherlands

18 Fondazione Poliambulanza Istituto Ospedaliero, Department of General Surgery, Brescia, Italy

19 Università degli Studi di Brescia, Department of Clinical and Experimental Sciences, Brescia, Italy

20 IEO European Institute of Oncology IRCCS, Division of Epidemiology and Biostatistics, Milan, Italy

21 Institute of Health and Society, University of Oslo, Department of Health Management and Health Economics, Oslo, Norway

22 Department of Surgery, School of Medicine, University of Jordan, Amman 11942, Jordan

23 Department of Surgery, University Hospital Southampton NHS Foundation Trust, Southampton, UK

24 Oslo University Hospital, The Intervention Centre, Department of Hepato-Pancreato-Biliary Surgery, and Institute of Clinical Medicine, University of Oslo, Oslo, Norway

**Corresponding authors**

Marc G Besselink, MD MSc PhD, [m.g.besselink@amsterdamUMC.nl](mailto:m.g.besselink@amsterdamUMC.nl)

Department of Surgery, Cancer Center Amsterdam

Amsterdam UMC, Location University of Amsterdam

De Boelelaan 1117, 1081 HV Amsterdam, The Netherlands

Mohammad Abu Hilal, MD PhD, [abuhilal9@gmail.com](mailto:abuhilal9@gmail.com)

Department of Surgery

School of Medicine, University of Jordan

Queen Rania Street, Amman 11942, Jordan

**Corresponding author during review process**

Julia E Menso, MD, [j.e.menso@amsterdamUMC.nl](mailto:j.e.menso@amsterdamUMC.nl)

**Article type:** Original article

**Key words:** Cost-analysis; cost-effectiveness; economic evaluation; quality adjusted life years; quality of life; minimally invasive pancreatoduodenectomy; robot-assisted surgery; pancreatic resection

**Disclosures:** JEM and MGB received a private donation to financially support the DIPLOMA-2x2 randomized trial about oncological outcome after RPD versus OPD. JEM, CLB, NdG, AMLHE, MAH, and MGB received research grants from Intuitive Foundation and the KWF Dutch Cancer Society for the investigator-initiated DIPLOMA-2x2 randomized trial. NdG, AMLHE, MAH, and MGB received a research grant from Intuitive Surgical Sarl. (Switzerland) for the investigator-initiated DIPLOMA-2 randomized trial.

**Authors contributions:** NdG, AMLHE, BB, UB, ORB, AC, FD, GF, SF, JvH, IHJTdH, MDH, BI, TK, BGK, DJL, MDPL, SJM, LM, IQM, MR, FS, and RFdW collected study data and revised the manuscript. HB and PM provided statistical advice and revised the manuscript. JEM, CLB, MA, GMWB, MAH, MGB, and AAF analyzed the study data and wrote the manuscript.

# Supplementary files

## Supplementary file 1: Details on the methods

**Eligibility criteria**

All patients undergoing RPD and OPD in the DIPLOMA-2 trial were included in this study. The original inclusion criteria of the DIPLOMA-2 trial were 1) adult patients (≥18 years), 2) with an indication for elective pancreatoduodenectomy for proven or suspected (pre)malignant lesions in the pancreatic head, distal bile duct, duodenum, and ampulla, 3) upfront resectable disease (prior to neoadjuvant therapy) and no signs of vascular involvement on preoperative CT-scan, 4) feasible for both MIPD and OPD, 5) fit to undergo surgery, and 6) written informed consent. The original exclusion criteria were 1) second malignancy requiring simultaneous resection, 2) indication/medical history of chronic pancreatitis, 3) any vascular involvement/distant metastases (including distant lymph nodes), 4) body mass index (BMI) ≥35 kg/m^2^, 5) pregnancy, and 6) participation in a study which interferes with the CCI score or time to functional recovery.

The eligibility criteria for center and surgeon participation in the DIPLOMA-2 trial were a center’s annual volume of >30 MIPD/year and surgeon’s total experience of >60 MIPD and >60 OPD.

**Data collection**

Data were prospectively collected including resources related to direct medical care. Direct medical care were days of hospital stay (including days at the medium care [MC]/intensive care [IC] unit) and the number/type of postoperative diagnostic and therapeutic procedures. Healthcare resources were quantified using online case report forms, questionnaires, and hospital information systems. Data on postoperative complications were collected from the 90-day follow-up case report form of the primary DIPLOMA-2 trial. As the primary DIPLOMA-2 trial included data up to 90 days postoperatively[1], additional data were collected between 3 to 6 months after surgery regarding readmission(s), MC/IC admission(s), unplanned hospital visits (i.e., emergency room (ER) and unplanned outpatient clinic visits), endoscopic, radiologic and surgical reinterventions, and blood transfusion. Any other missing values were collected. An additional questionnaire about routinely used instruments for RPD and OPD was filled out by all participating centers for quality assurance of intraoperative microcosts and to prevent an underestimation of these costs for both approaches.

**Clinical outcome and healthcare use**

Baseline characteristics included age (years), sex, BMI (kg/m^2^), American Society of Anesthesiologists (ASA) score[2], comorbidities (e.g., preoperative pancreatitis and diabetes), preoperative diagnosis, and fistula risk (based on main pancreatic duct [MPD] size and BMI). Intraoperative outcomes included conversion, operative time (incision to wound closure) with/without break between the resection and reconstruction phase (min), blood loss (mL), MPD size (mm), pancreatic texture, and vascular/additional organ resection. Histopathological findings included final diagnosis, radical (R0) resection rate based on the definition of the Royal College of Pathologists[3], and tumor size (mm). Postoperative outcomes included major complications (Clavien–Dindo grade ≥3)[4], procedure-specific complications based on definitions of the International Study Group for Pancreatic Surgery (ISGPS) and International Study Group for Liver Surgery (ISGLS) (i.e., POPF grade B/C[5], post-pancreatectomy hemorrhage [PPH] grade B/C [6], delayed gastric emptying [DGE] grade B/C [7], and bile leak grade B/C [8]), wound infection according to the Centers for Disease Control and Prevention (CDC) classification[9], organ failure, and total in-hospital/6-month mortality. Data on postoperative healthcare use included time to functional recovery (days)[1, 10], length of primary and total hospital stay (days), readmission, MC/IC admission, unplanned hospital visits, reinterventions, and blood transfusion. This study included new results on clinical outcome– compared to the DIPLOMA-2 trial – as this sub-analysis compared RPD only (instead of MIPD) to OPD with an extended 6-month (instead of 90-day) follow-up and additional data collection on previously missing values.

**Hospital costs**

No preoperative costs were included as these costs are expected to be equally distributed across the groups by randomization. Intraoperative costs were calculated based on fixed and additional costs per procedure. Fixed intraoperative costs included a standard set of instruments/materials per approach, and robot purchase/maintenance costs. The set of instruments/materials per approach was initially based on the standard institutional content as recently reported in the cost-analysis of Amsterdam UMC.[11] These sets were thereafter tailored to reflect the actual instruments and materials used per participating center, based on data from the additional survey on routinely used instruments/materials, to ensure an accurate estimation of intraoperative microcosts (including the number of surgeons present). In case of conversion from RPD to OPD, an additional OPD set was included in the intraoperative costs, minus the costs of two RPD needle drivers (normal and suture cut). The robot purchase/maintenance costs were based on the total lease costs of the robotic system at Amsterdam UMC in 2023 divided by the total number of robotic procedures in Amsterdam UMC in 2023 (€2,150 per RPD). In 2023, two robotic systems were available at Amsterdam UMC, corresponding to a total capacity of 10 operating days per week. Of these, 2.5 days were allocated to hepato-pancreato-biliary procedures, including one day dedicated to pancreatic surgeries. This indicates that pancreatic surgery accounted for 10% of the center’s total robotic system utilization. Additional intraoperative costs included operating room (OR) use, equipment, personnel (OR staff), and overhead per minute, based on the operative time and a previous study on OR prices for Dutch hospitals.[12] Postoperative costs included all hospital-related costs during 6 months postoperatively, including costs for primary hospital stay, readmission(s) (e.g., days at the ward, MC/IC admission[s], and reinterventions), and unplanned hospital visits. Reoperations were included in the analysis with fixed costs for either relaparotomy and relaparoscopy.

**Health-Related Quality of Life and Quality-Adjusted Life-Years**

Effects in the cost-effectiveness analysis were estimated as QALYs (i.e., one QALY equals one year in perfect health). The QALYs were estimated on patient level by multiplying time alive with HR-QoL – hereafter referred to as Health-Related Quality of Life (HR-QoL) index scores. The HR-QoL index scores were calculated with the individual EuroQol Five Dimensions Five-Level (EQ-5D-5L) health utility index scores on a scale from 0 (death) to 1 (perfect health)[13] using the Dutch algorithm (i.e., preferences of the general population in the Netherlands).

Separately, HR-QoL – hereafter referred to as HR-QoL descriptive values – was measured using the EQ-5D-5L (scores ranging from -0.45 to 1.00 with higher scores indicating better QoL)[13], and disease-specific questionnaires, i.e., the European Organization for Research and Treatment of Cancer (EORTC) core Quality of Life Questionnaire (QLQ-C30; scores ranging from 0 to 100 with higher scores indicating better functional status and QoL or worse symptom burden)[14] and the Pancreatic Cancer Module (QLQ-PAN26; scores ranging from 0 to 100 with higher scores indicating more severe symptoms)[15] at baseline (preoperatively) at 1-, 3-, and 6-months postoperatively. The 6-month questionnaire included additional questions about patient satisfaction regarding the scar tissue. In the EQ-5D-5L questionnaires, patients scored five aspects of health status, i.e., mobility, self-care, daily activities, pain or other discomforts, and anxiety/depression.[13] In the EORTC QLQ-C30 questionnaires, patients reported experienced symptoms related to their disease, e.g., physical, emotional, cognitive, and social functioning.[14] The QLQ-PAN26 questionnaires specifically assesses pancreatic cancer-related symptoms and treatment side-effects, e.g., pain, digestive symptoms, bowel habits, body image, and sexuality.[15]

**Statistical analysis**

Analyses were performed based on the modified intention-to-treat (mITT) principle, comprising patients in the group to which they were randomized. Patients with preoperatively diagnosed tumor progression leading to vascular involvement or metastasized disease on repeated imaging were excluded and replaced as they did not undergo surgery. Categorical data were analyzed with the Pearson’s chi-squared test and presented as frequencies with percentages. Normally distributed data were analyzed using the t-test and presented as means with standard deviations. Non-normally distributed continuous data were analyzed with the Mann-Whitney U test and expressed as medians with interquartile ranges.

Three analyses were performed: 1) total hospital costs, 2) QALYs, and 3) cost-effectiveness. The first analysis compared mean total hospital costs (euro) of RPD versus OPD with the t-test using non-parametric, bias-corrected and accelerated bootstrapping with 5,000 samples, reported as means with bias-corrected and accelerated (BCa) 95% confidence intervals (CIs). Sensitivity analyses were performed to assess the impact of 1) robot purchase/maintenance costs by excluding these costs, 2) diagnosis by excluding patients with preoperative diagnosis pancreatic ductal adenocarcinoma (PDAC), as PDAC is associated with a lower fistula risk – due to a fibrotic gland and dilated MPD[16] – and thus potentially lower hospital costs, 3) diagnosis by excluding patients with another preoperative diagnosis than PDAC (i.e., in patients with PDAC only), and 4) fistula risk by excluding patients with high preoperative POPF risk, based on MPD <3 mm and BMI >25 kg/m^2^).[16, 17]

In the second analysis, QALYs were calculated with the HR-QoL index scores (EQ-5D-5L) and estimated at patient level using the area under the curve. The HR-QoL index scores were analyzed with 1) a complete case analysis and 2) after mean imputation for baseline values[18], an EQ-5D-5L health utility index score of zero for the remaining follow‐up of deceased patients, and non-bootstrapped multiple imputation with chained equation for the remaining missing values, based on the stratification factors (i.e., diagnosis PDAC and fistula risk), preoperative comorbidity, preoperative ASA score, HR-QoL at baseline (and follow-ups preceeding the imputation time, e.g., HR-QoL at baseline and 1 month was used for imputation at 3 months and HR-QoL at baseline, 1 month and 3 months was used for imputation at 6 months), and postoperative costs.[19] Due to differences in baseline HR-QoL index score, the QALY analysis was repeated with post-hoc covariate adjustment (i.e., Manca correction).[20, 21] Separately – to add granularity to the analysis of HR-QoL index scores – HR-QoL descriptive values (EQ-5D-5L, QLQ-C30 and QLQ-PAN26) with RPD versus OPD were compared using longitudinal analysis of covariance, presented as means and mean differences with 95% CIs. The linear mixed model included a random intercept at patient level and fixed effects for HR-QoL at baseline, treatment group (RPD vs OPD), time (i.e., 1, 3, or 6 months postoperatively), treatment-by-time interaction, and the stratification factors[22] (i.e., diagnosis PDAC and fistula risk). Missing data at baseline were handled with mean imputation, as appropriate for RCTs.[18] Terminal intercurrent events for the EQ-5D-5L descriptives scores were handled using a composite strategy i.e., health utility index score of zero for deceased patients, conform the QALY analysis), while intercurrent events for QLQ-C30 and QLQ-PAN26 were handled using a hypothetical strategy under the missing at random assumption using full-information maximum likelihood estimation by the linear mixed model.[23] The HR-QoL descriptive values in both groups were visualized in figures per clinically relevant domain for all three questionnaires.

The third analysis estimated cost-effectiveness by taking the difference in total hospital costs between RPD and OPD divided by the difference in QALYs. Uncertainty was assessed using bootstrapping with replacement in 1,000 iterations. Multiple imputation by chained equations was performed for missing values. Thereafter, costs and QALYs were derived for both groups and presented as means with 95% CIs. Cost-effectiveness was reported as the probability that the ICER falls below the Dutch WTP threshold at €80.000 (i.e., how many estimated ICERs were below the WTP threshold among the 1,000 iterations). The cost-effectiveness with covariate adjustment in both groups was visualized in a cost-effectiveness plane with each dot representing one bootstrapped ICER and in a cost-effectiveness acceptability curve (CEAC) to assess the probability of cost-effectiveness of RPD and OPD per additional QALY using different WTP thresholds, increasing the results’ applicability to other countries. Sensitivity analyses – corrected for the same baseline imbalances – assessed the impact of 1) mortality by excluding all deceased patients (i.e., survivors only), 2) cause of death by excluding patients with non-complication related mortality (i.e., survivors and patients with complication-related mortality only), 3) annual volume by excluding low-volume centers (i.e., high-volume centers with >30 RPD/year only) and 4) by excluding high-volume centers (i.e., low-volume centers with ≤30 RPD/year only), and 5) fistula risk by excluding patients with low-risk for POPF (i.e., high-risk patients with MPD <3 mm and/or BMI >25 kg/m^2^ only) and 6) by excluding patients with high-risk for POPF (i.e., low-risk patients with MPD >3 mm and BMI ≤25 kg/m^2^ only). The HR-QoL were presented over time and the QALYs were calculated based on the AUC at patient level using imputation with repeated measures and covariate adjustment.

Statistical analyses were performed with SPSS software version 28 for Windows (IBM SPSS Statistics, Armonk, NY, USA) for clinical outcome and hospital costs, R studio version 4.5.2 (R Foundation for Statistical Computing) for HR-QoL descriptive values, and STATA version 19.5 (StatCorp LLC, College Station) for HR-QoL index scores and cost-effectiveness. A P-value below 0.05 was considered statistically significant.

## Supplementary file 2: Details on the results

**Health-Related Quality of Life and Quality-Adjusted Life-Years**

For the EQ-5D-5L questionnaires, responsiveness at baseline was comparable between RPD and OPD (86% vs 87%), whereas fewer patients responded at 6 months postoperatively in the RPD group (68% vs 79%). The complete case analysis showed a non-significantly better HR-QoL index score at baseline in the RPD group (0.84 vs 0.80; Δ0.04 [-0.001 to 0.08]). The HR-QoL was comparable at 1 month (0.79 vs 0.76; Δ0.04 [-0.01 to 0.08]) and 3 months after surgery (0.84 vs 0.83; Δ0.01 [-0.03 to 0.04]) and HR-QoL at 6 months was non-significantly worse after RPD (0.80 vs 0.85; Δ-0.04 [-0.10 to 0.01]). After mean imputation for missing baseline values and a health utility index score of zero for deceased patients, the HR-QoL index score was still comparable at 1 month (0.75 vs 0.74; Δ0.01 [-0.05 to 0.08]) and 3 months after surgery (0.78 vs 0.81; Δ-0.04 [-0.10 to 0.03]), but now with a worse HR-QoL at 6 months in the RPD group (0.71 vs 0.79; Δ-0.09 [ -0.17 to -0.005]). After non-bootstrapped multiple imputation, no data were missing. This analysis showed similar baseline HR-QoL index score for both groups with still comparable HR-QoL at 1 month (0.74 vs 0.73; Δ0.01 [-0.06 to 0.07]) and 3 months after surgery (0.77 vs 0.81; Δ-0.03 [-0.09 to 0.03]) and a worse HR-QoL at 6 months in the RPD group (0.72 vs 0.79; Δ-0.07 [-0.16 to -0.003]; Figure 1).

When stratifying by annual volume, HR-QoL index scores at 6 months remained worse after RPD in high-volume centers (n=122, 3 centers; 0.74 vs 0.85, Δ-0.11 [-0.21 to -0.01]), but were now comparable in low-volume centers (n=146, 11 centers; 0.70 vs 0.76, Δ-0.06 [-0.16 to 0.05]; Supplementary figure 3 and 4, respectively). When stratifying by POPF risk, the HR-QoL at 6 months became comparable in high-risk patients (n=170; 0.73 vs 0.76, Δ-0.03 [-0.13 to 0.06]), whereas in low-risk patients HR-QoL at 6 months after surgery remained worse after RPD (n=98; 0.70 vs 0.85, Δ-0.15 [-0.26 to -0.04]; Supplementary figure 5 and 6, respectively).

**Cost-effectiveness**

No statistically significant differences were found between groups for the separate EQ-5D-5L descriptive values per domain up to 3 months postoperatively. At 6 months postoperatively, mobility (0.12 vs 0.08, P=0.058), self-care (0.11 vs 0.08, P=0.090) and anxiety/depression (0.14 vs 0.10, P=0.051) were non-significantly worse in the RPD group, whereas usual activities (0.14 vs 0.09, P=0.031), pain/discomfort (0.16 vs 0.10, P=0.010), and total sum score (0.26 vs 0.18, P=0.004) were significantly worse after RPD (Supplementary table 5). Details on HR-QoL descriptive values based on the QLQ-C30 and QLQ-PAN26 questionnaires are presented per domain in Supplementary table 6–8.

When stratifying by annual volume, the ICER in high‑volume centers (n=122) was 6.011 (QALYs Δ−0.024, incremental cost −€143, 34% probability of RPD being cost‑effective), while OPD dominated in low‑volume centers (n=146; QALYs Δ−0.021, incremental cost €6,123; 13% probability of RPD being cost‑effective; Supplementary figures 9–10). When stratifying by POPF risk, RPD was still dominated by OPD in high‑risk (n=170; QALYs Δ−0.009, incremental cost €2,199, 30% probability of RPD being cost‑effective) and low‑risk patients (n=98; incremental cost €3,273; QALYs Δ−0.033; 22% probability of RPD being cost‑effective; Supplementary figures 11–12).

## Supplementary table 1: CHEERS 2022 checklist

| **Topic** | **No.** | **Item** | **Location where item is reported** |
| --- | --- | --- | --- |
| **Title** |  |  |  |
|  | 1 | Identify the study as an economic evaluation and specify the interventions being compared. | Title page |
| **Abstract** |  |  |  |
|  | 2 | Provide a structured summary that highlights context, key methods, results, and alternative analyses. | Abstract |
| **Introduction** |  |  |  |
| **Background and objectives** | 3 | Give the context for the study, the study question, and its practical relevance for decision making in policy or practice. | Introduction, last paragraph |
| **Methods** |  |  |  |
| **Health economic analysis plan** | 4 | Indicate whether a health economic analysis plan was developed and where available. | Supplementary file 1 |
| **Study population** | 5 | Describe characteristics of the study population (such as age range, demographics, socioeconomic, or clinical characteristics). | Supplementary file 1 |
| **Setting and location** | 6 | Provide relevant contextual information that may influence findings. | Methods, DIPLOMA-2 trial  Supplementary file 1 |
| **Comparators** | 7 | Describe the interventions or strategies being compared and why chosen. | Methods, DIPLOMA-2 trial |
| **Perspective** | 8 | State the perspective(s) adopted by the study and why chosen. | Supplementary file 1 |
| **Time horizon** | 9 | State the time horizon for the study and why appropriate. | Methods, DIPLOMA-2 trial  Supplementary file 1 |
| **Discount rate** | 10 | Report the discount rate(s) and reason chosen. | Methods, Hospital costs  Supplementary file 1 |
| **Selection of outcomes** | 11 | Describe what outcomes were used as the measure(s) of benefit(s) and harm(s). | Methods, Outcomes,  Hospital costs  Supplementary file 1 |
| **Measurement of outcomes** | 12 | Describe how outcomes used to capture benefit(s) and harm(s) were measured. | Not reported |
| **Valuation of outcomes** | 13 | Describe the population and methods used to measure and value outcomes. | Methods, Hospital costs  Supplementary file 1 |
| **Measurement and valuation of resources and costs** | 14 | Describe how costs were valued. | Methods, Hospital costs  Supplementary file 1 |
| **Currency, price date, and conversion** | 15 | Report the dates of the estimated resource quantities and unit costs, plus the currency and year of conversion. | Methods, Hospital costs  Supplementary file 1 |
| **Rationale and description of model** | 16 | If modelling is used, describe in detail and why used. Report if the model is publicly available and where it can be accessed. | Not applicable |
| **Analytics and assumptions** | 17 | Describe any methods for analysing or statistically transforming data, any extrapolation methods, and approaches for validating any model used. | Not reported |
| **Characterising heterogeneity** | 18 | Describe any methods used for estimating how the results of the study vary for subgroups. | Not reported |
| **Characterising distributional effects** | 19 | Describe how impacts are distributed across different individuals or adjustments made to reflect priority populations. | Not applicable |
| **Characterising uncertainty** | 20 | Describe methods to characterise any sources of uncertainty in the analysis. | Supplementary file 1 |
| **Approach to engagement with patients and others affected by the study** | 21 | Describe any approaches to engage patients or service recipients, the general public, communities, or stakeholders (such as clinicians or payers) in the design of the study. | Not reported |
| **Results** |  |  |  |
| **Study parameters** | 22 | Report all analytic inputs (such as values, ranges, references) including uncertainty or distributional assumptions. | Not applicable |
| **Summary of main results** | 23 | Report the mean values for the main categories of costs and outcomes of interest and summarise them in the most appropriate overall measure. | Table 2 and Figure 3 |
| **Effect of uncertainty** | 24 | Describe how uncertainty about analytic judgments, inputs, or projections affect findings. Report the effect of choice of discount rate and time horizon, if applicable. | Not reported |
| **Effect of engagement with patients and others affected by the study** | 25 | Report on any difference patient/service recipient, general public, community, or stakeholder involvement made to the approach or findings of the study | Not reported |
| **Discussion** |  |  |  |
| **Study findings, limitations, generalisability, and current knowledge** | 26 | Report key findings, limitations, ethical or equity considerations not captured, and how these could affect patients, policy, or practice. | Discussion, paragraphs 1-6 |
| **Other relevant information** |  |  |  |
| **Source of funding** | 27 | Describe how the study was funded and any role of the funder in the identification, design, conduct, and reporting of the analysis | Title page, Disclosures |
| **Conflicts of interest** | 28 | Report authors conflicts of interest according to journal or International Committee of Medical Journal Editors requirements. | Title page, Disclosures |

Legend supplementary table 1: Reference: Husereau D, Drummond M, Augustovski F, et al. Consolidated Health Economic Evaluation Reporting Standards 2022 (CHEERS 2022) Explanation and Elaboration: A Report of the ISPOR CHEERS II Good Practices Task Force. Value Health 2022;25. <doi:10.1016/j.jval.2021.10.008>.

## Supplementary table 2: Unit costs

| **Resource** | **Unit** | **Costs (euro, 2023)** | **Reference** |
| --- | --- | --- | --- |
| **Admission** |  |  |  |
| General ward stay | Day | 668,47 | DCM |
| MC/IC stay | Day | 2830,63 | DCM |
| **Outpatient hospital care** |  |  |  |
| Average unplanned visit costs based on:  Outpatient clinic visit | Visit | 124,56 | DCM |
| Emergency department visit | Visit | 267,80 | DCM |
| **Endoscopic reinterventions** |  |  |  |
| Endoscopic NGT placement | Procedure | 961,34 | CL |
| Cortrak feeding tube placement | Procedure | 480,67 | CL |
| Colonoscopy | Procedure | 968,41 | CL |
| Endoscopic ultrasound | Procedure | 847,03 | CL |
| Endoscopic sclerotherapy | Procedure | 1222,31 | CL |
| Bronchoscopy | Procedure | 962,50 | CL |
| Percutaneous endoscopic gastrostomy | Procedure | 1443,96 | CL |
| Video Assisted Thoracoscopic Surgery, drainage | Procedure | 3267,91 | CL |
| Gastroscopy | Procedure | 723,43 | CL |
| **Radiological reinterventions** |  |  |  |
| Average drainage costs based on: |  |  |  |
| Ultrasound-guided drainage | Procedure | 1200,46 | CL |
| CT-guided drainage | Procedure | 883,35 | CL |
| X-ray-guided PTC drainage | Procedure | 1049,34 | CL |
| Ultrasound-guided PTC drainage | Procedure | 1200,46 | CL |
| Vascular stent placement | Procedure | 2119,47 | CL |
| Vascular embolization | Procedure | 2119,47 | CL |
| Diagnostic angiography | Procedure | 187,71 | CL |
| Central venous catheter placement | Procedure | 750,57 | CL |
| **Surgical reinterventions** |  |  |  |
| Relaparoscopy for complication | Procedure | 6857,17 | CL |
| Relaparotomy for complication | Procedure | 5768,57 | CL |
| **Intraoperative costs** |  |  |  |
| Cost operating room (one surgeon, housing, equipment, personnel, overhead) | Minute | 11,52 | ORC |
| Cost operating room (two surgeons, housing, equipment, personnel, overhead) | Minute | 14,86 | ORC |
| Surgical set, open (or conversion)* | Set | 2126,23 | ORC |
| Surgical set, robot* | Set | 4201,92 | ORC |
| Robot purchase/maintenance | Usage cost | 2150,00 | CL |
| **Other** |  |  |  |
| Blood transfusion, erythrocytes | Bag | 446,20 | CL |

Legend supplementary table 2: Costs were derived from the Dutch costing manual (DCM)[24], AMC cost ledger (2018), ODV cost ledger (2023; CL), and Patel et al. (2022; ORC)[12], and indexed to 2023. *Mean prices of surgical sets are shown. Prices were calculated separately for each center based on the specific instruments used, and the per-procedure costs for RPD and OPD were determined accordingly.

## Supplementary table 3: Intraoperative and histopathological outcomes in patients undergoing RPD versus OPD

| **Intraoperative outcomes** | **RPD**  **(n=170)** | **OPD**  **(n=98)** | **P-value** |
| --- | --- | --- | --- |
| Conversion | 14 (8.2%) | NA | NA |
| Operative time incl break (min) | 336 (280–392) | 292 (228–345) | **<0.001** |
| Operative time excl break (min)  *Missing* | 303 (260–365)  *4* | 284 (215–330)  *4* | **<0.001** |
| Blood loss (mL)  *Missing* | 188 (100–300) | 300 (123–513) | **<0.001** |
| Small MPD ≤3 mm  *Missing* | 92 (54.1%)  *0* | 49 (51.0%)  *2* | 0.629 |
| Soft pancreas | 115 (67.6%) | 68 (69.4%) | 0.768 |
| Vascular resection | 6 (3.5%) | 5 (5.1%) | 0.532 |
| Additional organ resection | 4 (2.4%) | 1 (1.0%) | 0.437 |
| **Histopathological outcomes** |  |  |  |
| Final diagnosis PDAC  *Missing* | 52 (30.8%)  *1* | 26 (26.8%)  *1* | 0.494 |
| Final diagnosis any type of cancer  *Missing* | 131 (77.5%)  *1* | 66 (68.8%)  *2* | 0.189 |
| R0-resection rate* | 33 (63.5%) | 13 (50.0%) | 0.255 |
| Tumor size (mm)  *Missing* | 25 (18–34)  *10* | 22 (15–30)  *4* | **0.034** |

Legend supplementary table 3: Values are presented in medians with interquartile ranges and frequencies with percentages. RPD, robot-assisted pancreatoduodenectomy; OPD, open pancreatoduodenectomy; MPD, main pancreatic duct; PDAC, pancreatic ductal adenocarcinoma; NA, not applicable. *In patients with PDAC only.

## Supplementary table 4: EQ-5D-5L domains from baseline to 6 months postoperatively

| **EQ5D scale** | **RPD** | | **OPD** | | **Difference (95% CI)** | | **P value** |
| --- | --- | --- | --- | --- | --- | --- | --- |
| **Total (sum) score** |  |  |  |  |  |  |  |
| Baseline | 0.16 | (0.13 to 0.19) | 0.17 | (0.14 to 0.21) |  |  |  |
| 1 month | 0.23 | (0.19 to 0.27) | 0.23 | (0.18 to 0.28) | 0.00 | (–0.05 to 0.06) | 0.915 |
| 3 months | 0.18 | (0.14 to 0.22) | 0.15 | (0.11 to 0.20) | 0.03 | (–0.03 to 0.09) | 0.293 |
| 6 months | 0.26 | (0.22 to 0.30) | 0.18 | (0.13 to 0.22) | 0.08 | (0.03 to 0.14) | **0.004** |
| **Mobility** |  |  |  |  |  |  |  |
| Baseline | 0.05 | (0.02 to 0.08) | 0.04 | (0.01 to 0.07) |  |  |  |
| 1 month | 0.06 | (0.03 to 0.09) | 0.06 | (0.03 to 0.09) | 0.00 | (–0.04 to 0.04) | 0.971 |
| 3 months | 0.06 | (0.04 to 0.09) | 0.05 | (0.02 to 0.08) | 0.01 | (–0.03 to 0.05) | 0.587 |
| 6 months | 0.12 | (0.09 to 0.15) | 0.08 | (0.05 to 0.12) | 0.04 | (0.00 to 0.08) | 0.058 |
| **Self-care** |  |  |  |  |  |  |  |
| Baseline | 0.04 | (0.01 to 0.07) | 0.03 | (0.00 to 0.06) |  |  |  |
| 1 month | 0.05 | (0.02 to 0.08) | 0.05 | (0.01 to 0.08) | 0.00 | (–0.04 to 0.04) | 0.923 |
| 3 months | 0.05 | (0.02 to 0.08) | 0.04 | (0.01 to 0.07) | 0.01 | (–0.03 to 0.05) | 0.632 |
| 6 months | 0.11 | (0.08 to 0.14) | 0.08 | (0.04 to 0.11) | 0.03 | (–0.01 to 0.07) | 0.090 |
| **Usual activities** |  |  |  |  |  |  |  |
| Baseline | 0.06 | (0.03 to 0.09) | 0.05 | (0.02 to 0.08) |  |  |  |
| 1 month | 0.09 | (0.07 to 0.12) | 0.09 | (0.06 to 0.13) | 0.00 | (–0.04 to 0.04) | 0.968 |
| 3 months | 0.08 | (0.05 to 0.11) | 0.06 | (0.03 to 0.10) | 0.02 | (–0.02 to 0.06) | 0.427 |
| 6 months | 0.14 | (0.11 to 0.17) | 0.09 | (0.06 to 0.13) | 0.04 | (0.00 to 0.09) | **0.031** |
| **Pain/discomfort** |  |  |  |  |  |  |  |
| Baseline | 0.08 | (0.05 to 0.11) | 0.08 | (0.05 to 0.11) |  |  |  |
| 1 month | 0.09 | (0.07 to 0.12) | 0.09 | (0.06 to 0.12) | 0.00 | (–0.04 to 0.04) | 0.842 |
| 3 months | 0.09 | (0.06 to 0.12) | 0.07 | (0.03 to 0.10) | 0.02 | (–0.02 to 0.06) | 0.279 |
| 6 months | 0.16 | (0.13 to 0.18) | 0.10 | (0.07 to 0.14) | 0.05 | (0.01 to 0.09) | **0.010** |
| **Anxiety/depression** |  |  |  |  |  |  |  |
| Baseline | 0.09 | (0.05 to 0.12) | 0.07 | (0.04 to 0.09) |  |  |  |
| 1 month | 0.08 | (0.05 to 0.11) | 0.08 | (0.04 to 0.12) | 0.00 | (–0.05 to 0.04) | 0.896 |
| 3 months | 0.08 | (0.05 to 0.11) | 0.07 | (0.03 to 0.10) | 0.01 | (–0.03 to 0.05) | 0.574 |
| 6 months | 0.14 | (0.11 to 0.17) | 0.10 | (0.06 to 0.13) | 0.04 | (0.00 to 0.08) | 0.051 |

Legend supplementary table 4: Values are means with 95% confidence intervals (CIs). The HR-QoL descriptive values were adjusted for HR-QoL at baseline, treatment group, time, treatment-by-time interaction, and the stratification factors (i.e., diagnosis PDAC and fistula risk). RPD, robot-assisted pancreatoduodenectomy; OPD, open pancreatoduodenectomy.

## Supplementary table 5: EORTC QLQ-C30 functional scales from baseline to 6 months postoperatively

| **Functional scales** | **RPD** | | **OPD** | | | **Difference (95% CI)** | | | **P-value** |
| --- | --- | --- | --- | --- | --- | --- | --- | --- | --- |
| **Physical** |  |  | |  |  | |  |  |  |
| Baseline | 83 | (81 to 86) | | 81 | (77 to 84) | |  |  |  |
| 1 month | 69 | (65 to 73) | | 68 | (63 to 72) | | 1 | (–4 to 7) | 0.66 |
| 3 months | 78 | (74 to 82) | | 79 | (74 to 83) | | –1 | (–6 to 5) | 0.76 |
| 6 months | 78 | (74 to 82) | | 82 | (77 to 86) | | –4 | (–9 to 2) | 0.19 |
| **Role** |  |  | |  |  | |  |  |  |
| Baseline | 76 | (72 to 80) | | 72 | (66 to 77) | |  |  |  |
| 1 month | 52 | (46 to 57) | | 56 | (49 to 63) | | –4 | (–12 to 4) | 0.29 |
| 3 months | 68 | (62 to 73) | | 70 | (64 to 77) | | –3 | (–11 to 5) | 0.47 |
| 6 months | 71 | (65 to 77) | | 79 | (72 to 85) | | –8 | (–16 to 0) | 0.061 |
| **Emotional** |  |  | |  |  | |  |  |  |
| Baseline | 77 | (74 to 79) | | 72 | (68 to 76) | |  |  |  |
| 1 month | 78 | (74 to 81) | | 82 | (78 to 86) | | –4 | (–9 to 1) | 0.083 |
| 3 months | 85 | (81 to 88) | | 85 | (80 to 89) | | 0 | (–5 to 5) | 0.91 |
| 6 months | 81 | (77 to 84) | | 89 | (85 to 93) | | –9 | (–14 to –4) | **0.0008** |
| **Cognitive** |  |  | |  |  | |  |  |  |
| Baseline | 90 | (87 to 92) | | 86 | (83 to 89) | |  |  |  |
| 1 month | 82 | (78 to 86) | | 82 | (77 to 86) | | 0 | (–5 to 6) | 0.89 |
| 3 months | 86 | (82 to 90) | | 83 | (79 to 88) | | 2 | (–3 to 8) | 0.36 |
| 6 months | 85 | (81 to 89) | | 87 | (82 to 91) | | –2 | (–7 to 4) | 0.56 |
| **Social** |  |  | |  |  | |  |  |  |
| Baseline | 79 | (76 to 82) | | 77 | (72 to 81) | |  |  |  |
| 1 month | 68 | (64 to 73) | | 65 | (59 to 71) | | 4 | (–3 to 11) | 0.31 |
| 3 months | 75 | (70 to 80) | | 76 | (70 to 82) | | 0 | (–7 to 6) | 0.89 |
| 6 months | 77 | (72 to 82) | | 82 | (76 to 88) | | –5 | (–12 to 2) | 0.14 |

Legend supplementary table 5: Values are means with 95% confidence intervals (CIs). The HR-QoL descriptive values were adjusted for HR-QoL at baseline, treatment group, time, treatment-by-time interaction, and the stratification factors (i.e., diagnosis PDAC and fistula risk). RPD, robot-assisted pancreatoduodenectomy; OPD, open pancreatoduodenectomy.

## Supplementary table 6: EORTC QLQ-C30 symptom scales from baseline to 6 months postoperatively

| **Symptoms** | **RPD** | | **OPD** | | | **Difference (95% CI)** | | | **P-value** | |
| --- | --- | --- | --- | --- | --- | --- | --- | --- | --- | --- |
| **Fatigue** |  |  | |  |  | |  |  | |  |
| Baseline | 31 | (28 to 34) | | 33 | (29 to 38) | |  |  | |  |
| 1 month | 47 | (42 to 51) | | 42 | (36 to 47) | | 5 | (–1 to 12) | | 0.11 |
| 3 months | 36 | (32 to 41) | | 32 | (26 to 37) | | 5 | (–2 to 11) | | 0.17 |
| 6 months | 35 | (30 to 39) | | 32 | (27 to 38) | | 2 | (–4 to 9) | | 0.49 |
| **Nausea** |  |  | |  |  | |  |  | |  |
| Baseline | 10 | (8 to 13) | | 13 | (9 to 18) | |  |  | |  |
| 1 month | 15 | (11 to 19) | | 13 | (9 to 18) | | 2 | (–4 to 8) | | 0.52 |
| 3 months | 11 | (7 to 15) | | 10 | (5 to 14) | | 1 | (–4 to 7) | | 0.69 |
| 6 months | 10 | (6 to 14) | | 10 | (6 to 15) | | 0 | (–6 to 5) | | 0.88 |
| **Pain** |  |  | |  |  | |  |  | |  |
| Baseline | 17 | (13 to 20) | | 25 | (20 to 30) | |  |  | |  |
| 1 month | 26 | (22 to 31) | | 27 | (22 to 33) | | –1 | (–8 to 5) | | 0.74 |
| 3 months | 20 | (15 to 24) | | 20 | (15 to 25) | | 0 | (–7 to 6) | | 0.92 |
| 6 months | 22 | (17 to 26) | | 16 | (11 to 22) | | 6 | (–1 to 12) | | 0.10 |
| **Dyspnea** |  |  | |  |  | |  |  | |  |
| Baseline | 13 | (10 to 16) | | 16 | (11 to 20) | |  |  | |  |
| 1 month | 15 | (11 to 20) | | 18 | (13 to 24) | | –3 | (–9 to 4) | | 0.39 |
| 3 months | 14 | (9 to 18) | | 15 | (9 to 20) | | –1 | (–7 to 5) | | 0.72 |
| 6 months | 16 | (12 to 21) | | 11 | (6 to 17) | | 5 | (–2 to 11) | | 0.14 |
| **Insomnia** |  |  | |  |  | |  |  | |  |
| Baseline | 29 | (25 to 34) | | 34 | (28 to 40) | |  |  | |  |
| 1 month | 32 | (27 to 37) | | 28 | (22 to 34) | | 4 | (–3 to 12) | | 0.26 |
| 3 months | 24 | (19 to 29) | | 19 | (13 to 25) | | 5 | (–2 to 12) | | 0.16 |
| 6 months | 24 | (18 to 29) | | 17 | (11 to 24) | | 6 | (–1 to 14) | | 0.094 |
| **Appetite loss** |  |  | |  |  | |  |  | |  |
| Baseline | 22 | (18 to 26) | | 24 | (18 to 30) | |  |  | |  |
| 1 month | 36 | (30 to 42) | | 34 | (26 to 41) | | 2 | (–6 to 11) | | 0.57 |
| 3 months | 19 | (13 to 25) | | 24 | (17 to 31) | | –5 | (–13 to 4) | | 0.26 |
| 6 months | 21 | (15 to 27) | | 16 | (9 to 23) | | 5 | (–4 to 13) | | 0.30 |
| **Constipation** |  |  | |  |  | |  |  | |  |
| Baseline | 13 | (10 to 17) | | 14 | (10 to 19) | |  |  | |  |
| 1 month | 15 | (10 to 19) | | 15 | (10 to 20) | | –1 | (–7 to 6) | | 0.86 |
| 3 months | 12 | (8 to 16) | | 9 | (4 to 15) | | 3 | (–3 to 9) | | 0.40 |
| 6 months | 7 | (3 to 12) | | 9 | (3 to 14) | | –1 | (–8 to 5) | | 0.64 |
| **Diarrhea** |  |  | |  |  | |  |  | |  |
| Baseline | 14 | (11 to 18) | | 12 | (8 to 15) | |  |  | |  |
| 1 month | 16 | (11 to 21) | | 20 | (15 to 26) | | –4 | (–11 to 2) | | 0.20 |
| 3 months | 15 | (11 to 20) | | 13 | (8 to 19) | | 2 | (–4 to 9) | | 0.47 |
| 6 months | 17 | (13 to 22) | | 16 | (10 to 21) | | 2 | (–5 to 8) | | 0.62 |
| **Financial difficulties** |  |  | |  |  | |  |  | |  |
| Baseline | 4 | (2 to 6) | | 7 | (4 to 11) | |  |  | |  |
| 1 month | 3 | (0 to 6) | | 5 | (1 to 9) | | –2 | (–6 to 3) | | 0.43 |
| 3 months | 6 | (3 to 9) | | 5 | (1 to 8) | | 1 | (–3 to 6) | | 0.50 |
| 6 months | 9 | (6 to 12) | | 5 | (2 to 9) | | 4 | (0 to 8) | | 0.082 |

Legend supplementary table 6: Values are means with 95% confidence intervals (CIs). The HR-QoL descriptive values were adjusted for HR-QoL at baseline, treatment group, time, treatment-by-time interaction, and the stratification factors (i.e., diagnosis PDAC and fistula risk). RPD, robot-assisted pancreatoduodenectomy; OPD, open pancreatoduodenectomy.

## Supplementary table 7: EORTC QLQ-PAN26 domains from baseline to 6 months postoperatively

| **Domain** | **RPD** | | **OPD** | | | **Difference (95% CI)** | | | **P-value** | |
| --- | --- | --- | --- | --- | --- | --- | --- | --- | --- | --- |
| **Pancreatic pain** |  |  | |  |  | |  |  | |  |
| Baseline | 18 | (15 to 20) | | 23 | (19 to 27) | |  |  | |  |
| 1 month | 26 | (22 to 29) | | 26 | (22 to 30) | | –1 | (–6 to 4) | | 0.82 |
| 3 months | 21 | (17 to 24) | | 17 | (13 to 21) | | 3 | (–1 to 8) | | 0.16 |
| 6 months | 22 | (18 to 26) | | 18 | (13 to 22) | | 4 | (–1 to 9) | | 0.083 |
| **Digestive symptoms** |  |  | |  |  | |  |  | |  |
| Baseline | 25 | (21 to 29) | | 26 | (20 to 32) | |  |  | |  |
| 1 month | 40 | (34 to 45) | | 40 | (34 to 47) | | –1 | (–8 to 7) | | 0.86 |
| 3 months | 24 | (19 to 30) | | 26 | (19 to 32) | | –2 | (–9 to 6) | | 0.69 |
| 6 months | 23 | (17 to 28) | | 20 | (13 to 26) | | 3 | (–5 to 10) | | 0.52 |
| **Altered bowel habits** |  |  | |  |  | |  |  | |  |
| Baseline | 24 | (21 to 27) | | 26 | (22 to 31) | |  |  | |  |
| 1 month | 26 | (22 to 31) | | 23 | (17 to 28) | | 4 | (–3 to 10) | | 0.25 |
| 3 months | 28 | (23 to 32) | | 19 | (14 to 25) | | 8 | (2 to 15) | | 0.010 |
| 6 months | 29 | (24 to 34) | | 23 | (17 to 28) | | 6 | (0 to 13) | | 0.059 |
| **Hepatic symptoms** |  |  | |  |  | |  |  | |  |
| Baseline | 21 | (17 to 25) | | 25 | (20 to 30) | |  |  | |  |
| 1 month | 4 | (2 to 7) | | 6 | (3 to 9) | | –1 | (–5 to 2) | | 0.37 |
| 3 months | 5 | (2 to 7) | | 4 | (1 to 6) | | 1 | (–2 to 4) | | 0.51 |
| 6 months | 6 | (3 to 8) | | 3 | (0 to 5) | | 3 | (0 to 6) | | 0.061 |
| **Body image** |  |  | |  |  | |  |  | |  |
| Baseline | 18 | (15 to 21) | | 20 | (15 to 24) | |  |  | |  |
| 1 month | 22 | (18 to 27) | | 24 | (18 to 30) | | –2 | (–8 to 5) | | 0.64 |
| 3 months | 19 | (15 to 24) | | 17 | (11 to 22) | | 2 | (–4 to 9) | | 0.46 |
| 6 months | 19 | (14 to 24) | | 15 | (10 to 21) | | 4 | (–3 to 11) | | 0.24 |
| **Healthcare satisfaction** |  |  | |  |  | |  |  | |  |
| Baseline | 69 | (65 to 74) | | 74 | (68 to 79) | |  |  | |  |
| 1 month | 68 | (62 to 74) | | 74 | (66 to 81) | | –6 | (–14 to 3) | | 0.21 |
| 3 months | 66 | (60 to 72) | | 69 | (62 to 76) | | –3 | (–12 to 6) | | 0.49 |
| 6 months | 58 | (52 to 64) | | 61 | (54 to 69) | | –4 | (–12 to 5) | | 0.44 |
| **Sexual dysfunction** |  |  | |  |  | |  |  | |  |
| Baseline | 57 | (52 to 62) | | 47 | (40 to 54) | |  |  | |  |
| 1 month | 46 | (39 to 54) | | 52 | (44 to 60) | | –6 | (–16 to 4) | | 0.26 |
| 3 months | 57 | (49 to 64) | | 62 | (53 to 70) | | –5 | (–15 to 5) | | 0.33 |
| 6 months | 56 | (48 to 63) | | 73 | (64 to 81) | | –17 | (–27 to –7) | | **0.001** |

Legend supplementary table 7: Values are means with 95% confidence intervals (CIs). The HR-QoL descriptive values were adjusted for HR-QoL at baseline, treatment group, time, treatment-by-time interaction, and the stratification factors (i.e., diagnosis PDAC and fistula risk). RPD, robot-assisted pancreatoduodenectomy; OPD, open pancreatoduodenectomy.

## Supplementary figure 1: Flow-chart of patient inclusion

**Screening**

Screening

ening

**349 met the inclusion criteria for the DIPLOMA-2 trial and were asked for informed consent**

0 lost to follow-up

Analysis

Follow-Up

58 were eligible but not enrolled

- 48 declined participation
- 10 enrollment declined by physician: urgent surgery required

9 were excluded prior to surgery and replaced according to protocol

- 9 vascular involvement on repeated imaging

4 were excluded prior to surgery and replaced according to protocol

- 3 vascular involvement on repeated imaging
- 1 withdrew informed consent prior to surgery

**Enrollment**

Enrollment

98 patients allocated to OPD were included in

modified intention-to-treat analysis

170 patients allocated to RPD were included in

modified intention-to-treat analysis

190 allocated to minimally invasive PD

- 20 allocated to laparoscopic PD (**excluded**)
- 170 allocated to RPD (**included**)
  - 155 underwent RPD
  - 14 converted from RPD to OPD
  - 1 received exploration without resection due to metastases or locally advanced disease

0 lost to follow-up

98 allocated to OPD

- 97 underwent OPD
- 1 received exploration without resection due to metastases or locally advanced disease

**Allocation**

Allocation

**301 randomized**

**Analysis**

**Follow-up**

## Supplementary figure 2: Health-Related Quality of Life (EQ-5D-5L) over time in patients undergoing RPD versus OPD in the DIPLOMA-2 randomized trial up to 6 months excluding patients with disease progression-related mortality

Quality-adjusted life-years

Health-related quality of life

| Time (months) | Robot | Open |
| --- | --- | --- |
| 0 | 0.836129 | 0.806412 |
| 1 | 0.735332 | 0.732269 |
| 3 | 0.798781 | 0.813608 |
| 6 | 0.769052 | 0.823339 |

Legend supplementary figure 2: Health-Related Quality of Life (HR-QoL; Y-axis, utility) over time (X-axis, months) when excluding 12 patients with non-complication related mortality (n=256), based on the EuroQol Five Dimensions Five-Level (EQ-5D-5L) questionnaire, adjusted for HR-QoL at baseline. Quality-adjusted life-years (QALYs) were estimated as HR-QoL times time (i.e., area under the curve).

## Supplementary figure 3: Health-Related Quality of Life (EQ-5D-5L) over time in patients undergoing RPD versus OPD in the DIPLOMA-2 randomized trial up to 6 months in high-volume centers only

Quality-adjusted life-years

Health-related quality of life

| Time (months) | Robot | Open |
| --- | --- | --- |
| 0 | 0.842899 | 0.818135 |
| 1 | 0.825933 | 0.787117 |
| 3 | 0.811724 | 0.855373 |
| 6 | 0.737913 | 0.845659 |

Legend supplementary figure 3: Health-Related Quality of Life (HR-QoL; Y-axis, utility) over time (X-axis, months) in high-volume centers only (n=122, i.e., excluding 11 low-volume centers), based on the EuroQol Five Dimensions Five-Level (EQ-5D-5L) questionnaire, adjusted for HR-QoL at baseline. Quality-adjusted life-years (QALYs) were estimated as HR-QoL times time (i.e., area under the curve).

## Supplementary figure 4: Health-Related Quality of Life (EQ-5D-5L) over time in patients undergoing RPD versus OPD in the DIPLOMA-2 randomized trial up to 6 months in low-volume centers only

Quality-adjusted life-years

Health-related quality of life

| Time (months) | Robot | Open |
| --- | --- | --- |
| 0 | 0.832105 | 0.791999 |
| 1 | 0.672759 | 0.704832 |
| 3 | 0.756635 | 0.778558 |
| 6 | 0.702825 | 0.758336 |

Legend supplementary figure 4: Health-Related Quality of Life (HR-QoL; Y-axis, utility) over time (X-axis, months) in low-volume centers only (n=146, i.e., excluding 3 high-volume centers), based on the EuroQol Five Dimensions Five-Level (EQ-5D-5L) questionnaire, adjusted for HR-QoL at baseline. Quality-adjusted life-years (QALYs) were estimated as HR-QoL times time (i.e., area under the curve).

.

## Supplementary figure 5: Health-Related Quality of Life (EQ-5D-5L) over time in high-risk patients undergoing RPD versus OPD in the DIPLOMA-2 randomized trial up to 6 months

Quality-adjusted life-years

Health-related quality of life

| Time (months) | Robot | Open |
| --- | --- | --- |
| 0 | 0.840574 | 0.79266 |
| 1 | 0.742265 | 0.717653 |
| 3 | 0.789437 | 0.793038 |
| 6 | 0.730462 | 0.764743 |

Legend supplementary figure 5: Health-Related Quality of Life (HR-QoL; Y-axis, utility) over time (X-axis, months) in patients with high-risk for POPF only (n=170, i.e., excluding 98 low-risk patients), based on the EuroQol Five Dimensions Five-Level (EQ-5D-5L) questionnaire, adjusted for HR-QoL at baseline. Quality-adjusted life-years (QALYs) were estimated as HR-QoL times time (i.e., area under the curve).

## Supplementary figure 6: Health-Related Quality of Life (EQ-5D-5L) over time in low-risk patients undergoing RPD versus OPD in the DIPLOMA-2 randomized trial up to 6 months

Quality-adjusted life-years

Health-related quality of life

| Time (months) | Robot | Open |
| --- | --- | --- |
| 0 | 0.832167 | 0.822531 |
| 1 | 0.744775 | 0.752327 |
| 3 | 0.769741 | 0.828998 |
| 6 | 0.699474 | 0.845388 |

Legend supplementary figure 6: Health-Related Quality of Life (HR-QoL; Y-axis, utility) over time (X-axis, months) in patients with low-risk for POPF only (n=98, i.e., excluding 170 high-risk patients), based on the EuroQol Five Dimensions Five-Level (EQ-5D-5L) questionnaire, adjusted for HR-QoL at baseline. Quality-adjusted life-years (QALYs) were estimated as HR-QoL times time (i.e., area under the curve).

## Supplementary figure 7: Cost-effectiveness of RPD versus OPD in the DIPLOMA-2 randomized trial up to 6 months in survivors only

Cost-effectiveness plane

|  |  |
| --- | --- |
| -0.0101095 | 2647.83 |
| 0.0060202 | -686.96 |
| -0.0057075 | -6372.75 |
| 0.0008807 | 2822.25 |
| -0.0078297 | 7032.41 |
| 0.0053067 | 4433.33 |
| -0.0060201 | -2103.23 |
| -0.0044119 | -3879.83 |
| -0.0085635 | 3517.18 |
| -0.0041309 | -7428.52 |
| 0.0022262 | 4666.29 |
| -0.0053981 | -1833.43 |
| -0.0136508 | 11125 |
| 0.0003011 | -2209.04 |
| 0.0045896 | 4085.86 |
| -0.0066007 | 5268.94 |
| 0.0077472 | -6548.21 |
| -0.0166461 | -432.76 |
| 0.0099197 | 380.44 |
| -0.0164262 | 3836.4 |
| -0.0196841 | 5438.9 |
| 0.00482 | 6306.27 |
| -0.0012069 | 4196.19 |
| -0.0050773 | 6354.27 |
| -0.0047178 | 4016.38 |
| -0.0091153 | -3186.1 |
| 0.009077 | 5030.69 |
| -0.0070164 | 5641.24 |
| -0.0058096 | 5774.39 |
| 0.0075138 | 3059.73 |
| -0.0075939 | 7651.19 |
| 0.015728 | 3434.68 |
| 0.0051584 | -514.9 |
| -0.0011549 | 234.07 |
| 0.0021045 | 2969.82 |
| 0.0054763 | 4752.98 |
| -0.008385 | 5421 |
| 0.0009155 | 5607.73 |
| -0.006167 | 2607.23 |
| -0.0063796 | -4440.08 |
| -0.0032266 | 6035.04 |
| 0.0054514 | 9110.96 |
| -0.0067996 | 8091.48 |
| -0.0181797 | -5094.36 |
| -0.0150155 | -1046.36 |
| 0.0101914 | 8819.84 |
| 0.0059959 | 499 |
| -0.0049246 | 3399.1 |
| 0.0050286 | 3676.48 |
| -0.0040695 | 9015.49 |
| 0.0037279 | 2658.62 |
| -0.0125902 | -6467.95 |
| -0.0081411 | 3196 |
| -0.0012195 | 6728.68 |
| 0.0012549 | 5263.77 |
| -0.0116529 | 4383.21 |
| 0.0140437 | 2729.43 |
| -0.0022871 | -606.53 |
| -0.0030652 | 4302.38 |
| 0.010343 | 26.63 |
| -0.0139219 | 63.67 |
| -0.007249 | 7058.02 |
| 7.03e-06 | 301.75 |
| 0.0017932 | 3985.44 |
| 6.31e-05 | -12549.3 |
| -0.0105473 | -381 |
| 0.0006645 | 3399.94 |
| -0.0025418 | -2279.82 |
| -0.0033583 | -1442.94 |
| 0.0045066 | 6700.19 |
| -0.012685 | 3739.79 |
| -0.0119309 | 1548.43 |
| 0.0059642 | 3484.2 |
| 0.0045988 | -2071.55 |
| -0.0012683 | -1030.54 |
| 0.0008572 | 10361.6 |
| 0.0191117 | 4271.28 |
| -0.0001978 | 4223.75 |
| -0.0085064 | 10877.2 |
| -0.0054798 | -3565.68 |
| -0.0023778 | 3536.51 |
| -0.0062011 | -1486.04 |
| -0.0198546 | -2510.8 |
| 0.0251297 | 5178.34 |
| -0.0039346 | 3804.53 |
| 0.0030752 | -6703.96 |
| -0.0167938 | 3737.54 |
| -0.0037566 | 9173.52 |
| 0.005931 | 8918.76 |
| 0.0028242 | 1459.16 |
| 0.0001043 | 6070.41 |
| -0.0088147 | -2540.57 |
| 0.003154 | 4254.96 |
| 0.0069617 | 1342.7 |
| -0.008088 | 6060.09 |
| 0.0061579 | 3080.02 |
| -0.0056461 | -9244.83 |
| 0.0063552 | -206.18 |
| -0.0051315 | 2186.11 |
| -0.0066566 | 3666.06 |
| -0.0013566 | 4724.5 |
| -0.0088629 | 8096.46 |
| -0.004272 | 4882.46 |
| 0.0028157 | 2515.46 |
| -0.0021885 | 6458.65 |
| 0.0058007 | 8420.84 |
| 0.0137476 | 6790.83 |
| 0.0138907 | 7803.81 |
| -0.004169 | 2074.35 |
| -0.0140504 | 1675 |
| 0.0014593 | 7359.47 |
| -0.011337 | 1233.67 |
| -0.0013503 | -1110.8 |
| 0.0092596 | 1892.07 |
| -0.0066864 | -8786.2 |
| -0.0044142 | 4626.2 |
| 0.0006534 | 1648.86 |
| 0.0033635 | 8734.93 |
| -0.01355 | 2160.5 |
| 0.0013421 | 3857.28 |
| 0.000553 | 995.38 |
| 0.0046057 | 8481.18 |
| -0.0020982 | 8900.86 |
| -0.0072641 | 3434.6 |
| -0.0028527 | -6770.24 |
| -0.00484 | -3882.85 |
| -0.0095415 | -1340.38 |
| -0.0040558 | 6317.07 |
| -0.0039326 | -67.25 |
| -0.0142494 | -2734.07 |
| -0.0020449 | 5809.89 |
| -0.0079356 | 4397.04 |
| 0.0046622 | 4502.18 |
| 0.0010716 | 1449.31 |
| -0.0003222 | -3297.14 |
| -0.0110005 | 4263.76 |
| -0.0125531 | 2463.18 |
| 0.003647 | -6078.43 |
| -0.0076491 | -2235.42 |
| 0.010606 | 4961.65 |
| -0.0111825 | 5397.18 |
| -0.0033046 | 10120.9 |
| -0.010843 | -10615.5 |
| -0.0077485 | 4080.35 |
| -0.0007209 | 2740.37 |
| 0.0028193 | -1610.6 |
| 0.0005403 | 856.49 |
| -0.0090632 | 9741.2 |
| -0.0021518 | 3355.29 |
| -0.006635 | 3510.9 |
| 0.0013338 | -3988.6 |
| -0.0047597 | -1518.32 |
| -0.0100947 | 3907.78 |
| 0.0016165 | 3123.56 |
| 0.0059553 | 7040.79 |
| -0.0005408 | 5276.04 |
| 0.00242 | -993.57 |
| 0.0012656 | 1608 |
| 0.0044169 | 1977.72 |
| -0.0075705 | 4830.41 |
| 0.0001788 | -10476.4 |
| 0.0035859 | 5246.58 |
| 0.010149 | -1129.69 |
| -0.00405 | -4656.65 |
| 0.00319 | 4959.32 |
| -0.0076264 | 2561.24 |
| 0.0045094 | 9049.29 |
| -0.0033363 | 5019.35 |
| -0.0002282 | 9379.57 |
| -0.0063442 | 2753.04 |
| 0.000455 | -570.25 |
| -0.0127006 | -1321.81 |
| -0.004976 | 3462.92 |
| 0.0083985 | -412.51 |
| -0.0003186 | 3809.46 |
| 0.0010658 | 6526.91 |
| -0.0151833 | 5964.91 |
| 0.0111657 | 3953.35 |
| -0.0006718 | -2684.42 |
| -0.0144556 | 5975.06 |
| -0.0049766 | -1554.88 |
| 0.0011471 | 5173.76 |
| -0.0034535 | 4593.03 |
| 0.0027897 | -1295.12 |
| -0.0031336 | 1236.2 |
| -0.001481 | 6120.6 |
| -0.0134041 | 6821.67 |
| -0.0081072 | -3813.06 |
| -0.0109859 | -1861.82 |
| -0.0031466 | -5396.87 |
| -0.0144638 | -472.34 |
| -0.0112392 | 3.21 |
| 0.0041201 | 5900.53 |
| 0.0164624 | 912.35 |
| -0.0039992 | 97.54 |
| 0.0002438 | 2301.38 |
| -0.0025831 | 5146.95 |
| -0.0056743 | 4521.01 |
| -0.00261 | -3773.84 |
| -0.0040965 | 11732.9 |

Cost-effectiveness acceptability curves

Probability cost-effective

| Willingness to pay threshold | ROBOT SURGERY | OPEN SURGERY |
| --- | --- | --- |
| 0 | 0.267 | 0.733 |
| 10000 | 0.266 | 0.734 |
| 20000 | 0.263 | 0.737 |
| 30000 | 0.261 | 0.739 |
| 40000 | 0.258 | 0.742 |
| 50000 | 0.256 | 0.744 |
| 60000 | 0.251 | 0.749 |
| 70000 | 0.25 | 0.75 |
| 80000 | 0.247 | 0.753 |
| 90000 | 0.245 | 0.755 |
| 120000 | 0.245 | 0.755 |
| 130000 | 0.244 | 0.756 |
| 140000 | 0.245 | 0.755 |
| 150000 | 0.243 | 0.757 |
| 160000 | 0.24 | 0.76 |
| 170000 | 0.237 | 0.763 |

|  | EVPI |
| --- | --- |
| 0 | 773.29 |
| 10000 | 764.263 |
| 20000 | 755.397 |
| 30000 | 746.988 |
| 40000 | 739.026 |
| 50000 | 731.436 |
| 60000 | 724.526 |
| 70000 | 718.343 |
| 80000 | 712.769 |
| 90000 | 707.758 |
| 120000 | 696.026 |
| 130000 | 692.756 |
| 140000 | 689.858 |
| 150000 | 687.15 |
| 160000 | 685.219 |
| 170000 | 683.849 |

Legend supplementary figure 7: Upper figure: the cost-difference between RPD and OPD (Y-axis, euro) and the QALY-difference (X-axis). Lower figure: The probability of cost-effectiveness (Y-axis, rate from 0 to 1) for different willingness-to-pay thresholds (X-axis, euro).

## Supplementary figure 8: Cost-effectiveness of RPD versus OPD in the DIPLOMA-2 randomized trial up to 6 months excluding patients with disease progression-related mortality

Cost-effectiveness plane

|  |  |
| --- | --- |
| 0.0027994 | -4421.17 |
| -0.0185666 | 6511.39 |
| 0.0035447 | -2583.29 |
| -0.0230707 | 9797.95 |
| 0.0010712 | -1190.16 |
| -0.0256409 | 4860.21 |
| 0.0002317 | 3368.52 |
| -0.0233766 | 21.11 |
| -0.0085553 | 1814.54 |
| -0.0005251 | 3379.29 |
| -0.0132815 | 10493.5 |
| -0.0023727 | -974.75 |
| 0.0053948 | -1165.31 |
| -0.0057245 | 506.93 |
| -0.0187692 | 6247.54 |
| -0.0202673 | 2172.21 |
| -0.0037976 | 5842.56 |
| 0.0062285 | -6492.86 |
| -0.0124348 | 4202.23 |
| -0.0069914 | -984.73 |
| -0.0108507 | 3018.96 |
| -0.0226875 | 5781.75 |
| -0.0103522 | 2269.83 |
| -0.0121101 | -274.79 |
| -0.0097495 | 1897.46 |
| -0.01008 | 5649.96 |
| -0.0345928 | 8140.78 |
| -0.004955 | -988.38 |
| -0.0067935 | 7948.4 |
| -0.020494 | 2739.9 |
| -0.0178965 | 5099.52 |
| -0.0142447 | -1036.92 |
| -0.0173112 | 5233.33 |
| -0.0120366 | 1024.49 |
| -0.0156745 | -4059.56 |
| -0.0166538 | 9961.72 |
| -0.0040608 | -6242.72 |
| 0.0038158 | -3977.72 |
| -0.0503837 | 6000.51 |
| -0.006936 | -7710.99 |
| -0.0267097 | 6097.5 |
| 0.0098816 | 1472.42 |
| -0.0180888 | 9021.22 |
| -0.0304464 | 525.18 |
| -0.0238598 | 9763.14 |
| 0.0012745 | -4713.95 |
| -0.0097034 | -2551.2 |
| -0.0231828 | 4218.78 |
| -0.0087254 | 2583.14 |
| -0.0168299 | 3272.17 |
| 0.0063976 | -2185.66 |
| -0.0142364 | 4666.4 |
| -0.0078922 | 193.07 |
| -0.0206997 | -1420.49 |
| -0.0263107 | 1520.84 |
| -0.014259 | 8164.54 |
| -0.012419 | -2735.05 |
| -0.0121097 | 7056.07 |
| -0.0124955 | 4474.92 |
| -0.00737 | -4079.55 |
| -0.0121071 | 6983.12 |
| -0.0266755 | 4268.79 |
| 0.0018563 | -4060.01 |
| -0.0225811 | 351 |
| -0.0060815 | -2010.32 |
| -0.0335276 | 1723.72 |
| -0.0294068 | 7527.3 |
| -0.0228456 | 933.11 |
| -0.0024881 | 1785.67 |
| -0.0204214 | 4135.88 |
| -0.0281955 | 4872.93 |
| -0.0238669 | 6010.76 |
| -0.0240017 | 2666.61 |
| -0.0307042 | 2514.09 |
| -0.0145251 | -933.69 |
| -0.0324765 | 8549.64 |
| 0.0002957 | 5547.86 |
| -0.0212337 | 5365.23 |
| 0.0162112 | -8252.83 |
| -0.0307201 | 4471.23 |
| -0.028531 | 1695.17 |
| -0.0196781 | 2667.21 |
| -0.02464 | 4426.64 |
| -0.0214203 | 8057.66 |
| -0.0061761 | -2745.3 |
| -0.025881 | 9849.92 |
| -0.008814 | 2674.62 |
| -0.0020118 | -4655.11 |
| -0.0162263 | -129.24 |
| -0.0265228 | -295.93 |
| -0.0043237 | -6962.26 |
| -0.0365265 | 8571.48 |
| -0.0310306 | 5382.96 |
| 0.0008247 | -54.53 |
| -0.0421921 | 2287.77 |
| 0.0064394 | 3910.28 |
| -0.0068429 | 5354.41 |
| -0.0185036 | 5698.15 |
| -0.025605 | 1164.36 |
| -0.0065176 | 1243.43 |
| -0.017031 | 8122.44 |
| -0.0147329 | 6160.79 |
| -0.0195889 | 2430.32 |
| -0.0205299 | 414.12 |
| -0.0093704 | -2682.42 |
| -0.0558663 | 10027.2 |
| -0.0137661 | 3497.1 |
| -0.0021937 | -4161.07 |
| -0.0118171 | 3852.93 |
| -0.0106267 | -5038.46 |
| -0.0106457 | -473.18 |
| -0.0287599 | 1877.52 |
| -0.0241529 | 2829.53 |
| -0.021614 | 5304.66 |
| -0.0143295 | 4036.11 |
| -0.0277077 | 4584.83 |
| -0.0214597 | 6866.36 |
| -0.0169393 | 1379.24 |
| -0.0389417 | 12444.4 |
| -0.0072556 | 630.93 |
| -0.0031464 | 1232.96 |
| -0.0247777 | 5448.27 |
| -0.0184771 | -234.13 |
| -0.0173189 | 1572.23 |
| -0.0205319 | -3542.23 |
| 0.0131112 | 975.94 |
| -0.0300567 | 7457.28 |
| -0.0225402 | 783.05 |
| -0.0221415 | 6621.49 |
| -0.0338955 | 8699.96 |
| -0.0247952 | 1506.57 |
| 0.0018427 | 2302.43 |
| 0.0104116 | -2130.5 |
| 0.0065623 | 2568.54 |
| -0.0321553 | 8628.33 |
| -0.0189273 | 6154.97 |
| 0.0284835 | -13007.2 |
| -0.0409666 | 9090.15 |
| -0.0370774 | 6771.32 |
| -0.0096029 | 9458.87 |
| -0.0245107 | 2473.81 |
| -0.018476 | 4429.82 |
| -0.0201368 | 9827.85 |
| -0.0115366 | -1952.58 |
| 0.0054113 | 3576.63 |
| -0.013612 | 1096.81 |
| -0.009254 | 1994.46 |
| -0.0280435 | 3183.69 |
| -0.0028119 | -3156.35 |
| -0.0322107 | 4637.32 |
| -0.0170242 | -2092.54 |
| -0.0184823 | 4793.25 |
| 0.0022428 | 165.51 |
| -0.0143507 | 6413.59 |
| -0.0126805 | -3055.12 |
| -0.0462824 | 5541.54 |
| -0.0349356 | 11375.9 |
| -0.016409 | 4832.22 |
| -0.0002 | 7558.91 |
| 0.0119454 | -9720.65 |
| -0.0092866 | 3000.71 |
| -0.0133761 | 3546.42 |
| -0.0115368 | -7528.79 |
| -0.0106463 | -725.63 |
| -0.0279453 | -462.11 |
| -0.0088123 | -817.45 |
| -0.0240056 | 5644.56 |
| -0.0032119 | -924.94 |
| -0.0035922 | 3803.33 |
| -0.0156003 | 5469.95 |
| -0.0207254 | 303.85 |
| -0.0072563 | 317.35 |
| -0.0089219 | 4421.4 |
| -0.0203314 | -385.39 |
| -0.0402043 | 10665.9 |
| -0.0293943 | 4489.17 |
| -0.0193033 | 3280.16 |
| 0.00195 | 3101.87 |
| -0.0019887 | -7871.32 |
| -0.017253 | 8593.15 |
| -0.0177911 | 4590.8 |
| -0.0029386 | 1880.73 |
| -0.0104505 | 3792.62 |
| -0.0027146 | -688.65 |
| -0.0192121 | 7101.42 |
| -0.0084351 | 10369.2 |
| -0.0302781 | 9791.99 |
| 0.0077443 | -2641.11 |
| -0.0064669 | -848.24 |
| -0.0357176 | 5762.36 |
| -0.016981 | 5482.57 |
| -0.0093882 | 5479.58 |
| -0.0199411 | 5971.71 |
| -0.0080144 | 2939.74 |
| -0.030543 | 8534.67 |
| -0.0071111 | 4970.9 |
| -0.0042839 | -2011.9 |
| -0.013176 | 293.93 |
| -0.0102016 | 1909.95 |
| -0.0150004 | 3705.78 |

Cost-effectiveness acceptability curves

Probability cost-effective

| Willingness to pay threshold | ROBOT SURGERY | OPEN SURGERY |
| --- | --- | --- |
| 0 | 0.26 | 0.74 |
| 10000 | 0.253 | 0.747 |
| 20000 | 0.248 | 0.752 |
| 30000 | 0.239 | 0.761 |
| 40000 | 0.232 | 0.768 |
| 50000 | 0.228 | 0.772 |
| 60000 | 0.222 | 0.778 |
| 70000 | 0.213 | 0.787 |
| 80000 | 0.212 | 0.788 |
| 90000 | 0.209 | 0.791 |
| 120000 | 0.197 | 0.803 |
| 130000 | 0.196 | 0.804 |
| 140000 | 0.195 | 0.805 |
| 150000 | 0.193 | 0.807 |
| 160000 | 0.189 | 0.811 |
| 170000 | 0.187 | 0.813 |

|  | EVPI |
| --- | --- |
| 0 | 802.552 |
| 10000 | 787.784 |
| 20000 | 774.288 |
| 30000 | 761.909 |
| 40000 | 750.694 |
| 50000 | 740.303 |
| 60000 | 730.615 |
| 70000 | 722.183 |
| 80000 | 715.239 |
| 90000 | 708.865 |
| 120000 | 695.067 |
| 130000 | 691.784 |
| 140000 | 688.608 |
| 150000 | 685.703 |
| 160000 | 683.445 |
| 170000 | 681.45 |

Legend supplementary figure 8: Upper figure: the cost-difference between RPD and OPD (Y-axis, euro) and the QALY-difference (X-axis). Lower figure: The probability of cost-effectiveness (Y-axis, rate from 0 to 1) for different willingness-to-pay thresholds (X-axis, euro).

## Supplementary figure 9: Cost-effectiveness of RPD versus OPD in the DIPLOMA-2 randomized trial up to 6 months in high-volume centers only

Cost-effectiveness plane

|  |  |
| --- | --- |
| -0.0389284 | 2803.39 |
| -0.0150462 | -7689.61 |
| -0.0135779 | 2401.4 |
| -0.0052069 | 2573.88 |
| -0.0073154 | 848.89 |
| -0.0324134 | 5444.06 |
| -0.0460178 | -3494.89 |
| -0.0163442 | -12.12 |
| -0.0033139 | 2010.4 |
| -0.0325011 | -702.38 |
| -0.0138293 | 3648.23 |
| -0.023997 | 7896.69 |
| -0.0212469 | -1554.46 |
| -0.0201636 | 7863.51 |
| -0.0270359 | 2175.29 |
| -0.0361279 | 4097.49 |
| -0.0131899 | -508.34 |
| -0.0248526 | 4965.08 |
| -0.0129303 | -12050.2 |
| -0.044079 | 3089.27 |
| -0.0359753 | -472.53 |
| -0.0204957 | 2075.57 |
| -0.0075741 | 2836.15 |
| -0.0080829 | 5609.97 |
| -0.0130989 | -5939.51 |
| -0.013555 | -11972.3 |
| -0.0200873 | 1089.07 |
| -0.0196032 | -1748.08 |
| -0.0276104 | 4419.97 |
| -0.0308693 | 1846.27 |
| -0.0144779 | -930.46 |
| -0.0296539 | 57.81 |
| -0.0445702 | -6785.14 |
| -0.0606744 | 507.62 |
| -0.0366489 | 479.22 |
| -0.042289 | -13091.2 |
| -0.0221002 | 906.62 |
| -0.0171139 | 548.41 |
| -0.0148833 | 3263.18 |
| -0.0052033 | -5675.83 |
| -0.0288903 | -590.99 |
| -0.0350728 | -4208.16 |
| 0.0049507 | 4054.31 |
| -0.0195216 | 10273.2 |
| -0.031373 | 1883.53 |
| -0.0249028 | -2606.88 |
| -0.0518873 | -2076.13 |
| -0.0224446 | -1400.64 |
| -0.0122657 | 1702.63 |
| -0.0426077 | -13229.2 |
| -0.0173383 | -2522.89 |
| -0.0293844 | 2802.02 |
| -0.0104393 | -15977.7 |
| -0.0205764 | 8323.74 |
| -0.0010419 | 1841.73 |
| 0.0015349 | -4228.74 |
| -0.01829 | -8502.87 |
| -0.0297159 | 7222.1 |
| -0.0398031 | 2883.96 |
| -0.0253907 | 8022.17 |
| -0.0474776 | -1812.57 |
| -0.0281404 | 6937.06 |
| -0.030558 | 5914.29 |
| -0.0264417 | -11939.4 |
| -0.0172217 | -7791.34 |
| -0.039516 | 4965.96 |
| -0.0220214 | 121.96 |
| -0.0223272 | 2356.76 |
| -0.0104126 | 7783.17 |
| -0.0502749 | 780.69 |
| -0.0295964 | 4969.55 |
| -0.0304487 | -80.05 |
| -0.0308874 | -6784.12 |
| -0.0253332 | -2164.25 |
| -0.0211353 | -2053.48 |
| -0.035706 | 4261.29 |
| -0.0207918 | 1010.96 |
| -0.0066484 | 403.89 |
| -0.0302377 | 2762.42 |
| -0.0298654 | 6400.18 |
| -0.0194551 | 4898.79 |
| -0.0318255 | 83.05 |
| -0.0118301 | -821.61 |
| -0.0091145 | 1128 |
| -0.0243314 | 2115.24 |
| -0.0338237 | 2555.69 |
| -0.0252387 | 4689.89 |
| -0.0258624 | -4447.99 |
| -0.0299702 | -323.76 |
| -0.0066435 | -5754.38 |
| -0.0342046 | 3122.18 |
| -0.0199759 | -1027.47 |
| -0.0262819 | -2723.12 |
| -0.0172397 | 641.29 |
| -0.0321338 | 3658.8 |
| -0.0206633 | 3182.88 |
| 0.0166257 | -11737 |
| -0.0258952 | -1531.78 |
| -0.0676006 | 8378.82 |
| -0.0327036 | -8484.39 |
| -0.0504355 | 2568.82 |
| -0.0212116 | -2925.94 |
| -0.0306324 | 3359.73 |
| -0.0204458 | 1966.46 |
| 0.0004541 | -15296.2 |
| -0.0065177 | -3174.37 |
| -0.0269881 | -2705.04 |
| -0.0089993 | 4256.31 |
| -0.0274659 | 3095.92 |
| -0.0189126 | 6725.76 |
| -0.0351399 | 1268.12 |
| -0.0291963 | 5184 |
| -0.0093814 | 2070.79 |
| -0.0215788 | -11054.6 |
| -0.0202442 | 9498.47 |
| -0.0252583 | 5905.89 |
| -0.0205988 | -10633.6 |
| -0.0259897 | 4878.84 |
| -0.0204685 | -45.42 |
| -0.0130214 | -7215.26 |
| -0.0361719 | 6984.12 |
| -0.0128619 | -852.5 |
| -0.0384401 | -2528.78 |
| -0.0180682 | -7476.32 |
| -0.0185844 | -4101.29 |
| -0.0409039 | -6166.19 |
| -0.0225659 | -3277.99 |
| -0.0134889 | -3820.43 |
| -0.0258199 | -7207.19 |
| -0.0348248 | 6569.2 |
| -0.0155058 | -1730.23 |
| -0.0233321 | 4576.63 |
| -0.0222332 | 4998.15 |
| -0.017557 | -7530.04 |
| -0.0263932 | -11876 |
| -0.0202718 | 7414.87 |
| -0.0268111 | -6982.47 |
| -0.0124589 | -528.34 |
| -0.0149834 | -4821.94 |
| -0.0429316 | 4070.75 |
| -0.0194747 | -14472.3 |
| -0.0252907 | 2685.84 |
| -0.0181671 | -8979.49 |
| -0.0130676 | -3673.8 |
| -0.0001317 | -4930.89 |
| -0.0055894 | 1874.92 |
| -0.016102 | 5126.92 |
| -0.0139282 | 3715.98 |
| -0.0244501 | -1693.01 |
| -0.0231847 | -11692 |
| -0.0098547 | -11501.1 |
| -0.0198682 | 3517.35 |
| -0.0373741 | -4906.09 |
| -0.0054944 | 1423.62 |
| -0.0131181 | -19180 |
| -0.0159293 | 207.87 |
| -0.0260063 | -9097.79 |
| -0.011457 | -1006.22 |
| -0.034763 | 4416.53 |
| -0.0189921 | -12757.6 |
| -0.0368317 | 613.61 |
| -0.0194558 | 4209.28 |
| -0.0220731 | 1903.42 |
| -0.0186228 | -5434.68 |
| -0.0491724 | 4316.27 |
| -0.0219852 | -9294.43 |
| 0.0055085 | -6675.48 |
| -0.0341658 | 1381.57 |
| -0.0239482 | -4967.39 |
| -0.0250162 | 4981.5 |
| -0.0335173 | 6046.69 |
| -0.0089607 | -14659.9 |
| -0.0198286 | -3183.35 |
| -0.0129769 | -3787.39 |
| -0.0209133 | -4224.97 |
| -0.0030006 | -4848.24 |
| -0.0458182 | 481.12 |
| -0.0366071 | 9955.54 |
| -0.034157 | 2548.68 |
| -0.0401289 | 4782.13 |
| -0.0118098 | 5379.69 |
| -0.0174996 | -8061.39 |
| -0.020107 | -5864.79 |
| -0.032092 | 10699.7 |
| -0.0318103 | -1204.14 |
| -0.0054126 | -3457.56 |
| -0.06203 | 5175.51 |
| -0.0071769 | -36.3 |
| -0.0158373 | 2281.55 |
| 0.0026996 | -3935.8 |
| -0.0229985 | -713.23 |
| -0.0295745 | 2815.04 |
| -0.0226133 | -5506.86 |
| -0.020655 | -48.74 |
| -0.0218083 | -3812.77 |
| -0.0014457 | 5152.94 |
| -0.0092157 | -8704.19 |
| -0.0170082 | 979.95 |
| -0.0200047 | 5508.25 |
| -0.027535 | 6899.9 |

Cost-effectiveness acceptability curves

Probability cost-effective

| Willingness to pay threshold | ROBOT SURGERY | OPEN SURGERY |
| --- | --- | --- |
| 0 | 0.465 | 0.535 |
| 10000 | 0.446 | 0.554 |
| 20000 | 0.435 | 0.565 |
| 30000 | 0.418 | 0.582 |
| 40000 | 0.401 | 0.599 |
| 50000 | 0.389 | 0.611 |
| 60000 | 0.376 | 0.624 |
| 70000 | 0.355 | 0.645 |
| 80000 | 0.343 | 0.657 |
| 90000 | 0.333 | 0.667 |
| 120000 | 0.301 | 0.699 |
| 130000 | 0.29 | 0.71 |
| 140000 | 0.281 | 0.719 |
| 150000 | 0.269 | 0.731 |
| 160000 | 0.263 | 0.737 |
| 170000 | 0.254 | 0.746 |

|  | EVPI |
| --- | --- |
| 0 | 2382.09 |
| 10000 | 2284.07 |
| 20000 | 2189.76 |
| 30000 | 2099.89 |
| 40000 | 2014.77 |
| 50000 | 1934.71 |
| 60000 | 1857.22 |
| 70000 | 1784.15 |
| 80000 | 1715.35 |
| 90000 | 1649.28 |
| 120000 | 1467.6 |
| 130000 | 1413.21 |
| 140000 | 1362 |
| 150000 | 1313.25 |
| 160000 | 1267.79 |
| 170000 | 1223.74 |

Legend supplementary figure 9: Upper figure: the cost-difference between RPD and OPD (Y-axis, euro) and the QALY-difference (X-axis). Lower figure: The probability of cost-effectiveness (Y-axis, rate from 0 to 1) for different willingness-to-pay thresholds (X-axis, euro).

## Supplementary figure 10: Cost-effectiveness of RPD versus OPD in the DIPLOMA-2 randomized trial up to 6 months in low-volume centers only

Cost-effectiveness plane

|  |  |
| --- | --- |
| -0.0067587 | 6452.61 |
| -0.0147576 | 10140.8 |
| -0.0539009 | 9046.56 |
| -0.015244 | 1496.4 |
| -0.0088491 | 3183.21 |
| -0.0008602 | -735.49 |
| -0.0253644 | 7112.83 |
| -0.0617711 | 19556.4 |
| -0.0452267 | 178.07 |
| -0.0158564 | 6156.21 |
| 0.0054396 | 6886.68 |
| -0.0233766 | 12542.9 |
| -0.015848 | 2492.88 |
| -0.0081257 | 6984.76 |
| -0.0420661 | 12575.9 |
| -0.0358506 | 17465 |
| 0.0011042 | -5209.39 |
| -0.0037592 | -1485.75 |
| -0.0234069 | 15452 |
| -0.0400008 | 12287.7 |
| -0.0147832 | 11772.9 |
| 0.0111347 | 6992.11 |
| 0.0057804 | -4127.83 |
| -0.0372462 | 11677.9 |
| -0.010363 | 3724.17 |
| -0.0028615 | -720.38 |
| -0.0149106 | 640.89 |
| -0.0493797 | 5433.18 |
| -0.0365689 | 6094.01 |
| 0.01294 | -4417.93 |
| -0.0422297 | 10227.8 |
| 0.009084 | -6943.28 |
| -0.0181792 | -11430.9 |
| -0.0247781 | 3445.71 |
| -0.0372952 | 17841.1 |
| -0.0629667 | 18427.8 |
| -0.0054382 | 661.98 |
| -0.0102152 | 11892.2 |
| -0.0184721 | 14764.2 |
| -0.0037552 | -1335.79 |
| -0.0134796 | 6922.52 |
| -0.0320333 | 2966.94 |
| -0.0152169 | -106.89 |
| -0.0206467 | 3908.09 |
| -0.0133387 | 3534.48 |
| -0.0149129 | 10619 |
| 6.67e-05 | -2545.98 |
| -0.0097298 | -2735.92 |
| -0.0158848 | 1788.54 |
| -0.0165395 | 4662.77 |
| -0.0085968 | -2850.65 |
| 0.0078993 | 2585.72 |
| -0.0179874 | 11149.7 |
| -0.0149725 | 2740.99 |
| -0.0138958 | -167.54 |
| -0.0090069 | 8223.66 |
| -0.0251863 | 8842.79 |
| -0.0276939 | 325.79 |
| -0.0189767 | 5951.47 |
| -0.0206143 | 6409.51 |
| -0.0246352 | 7986.03 |
| -0.0208971 | 3039.89 |
| -0.0180301 | 11925.1 |
| -0.0058369 | 5720.43 |
| -0.0028836 | 4678.17 |
| -0.0422288 | 8559.13 |
| -0.0197244 | 7838.98 |
| -0.003104 | 6236.02 |
| -0.0336902 | 9267.09 |
| -0.0093724 | 4598.13 |
| 0.0078293 | -2816.9 |
| -0.0024009 | -8117.7 |
| -0.0459734 | 12758.1 |
| -0.0092236 | 9022.08 |
| -0.0284635 | 15839.9 |
| -0.0153779 | 9326.17 |
| 0.0047789 | -815.22 |
| -0.0416049 | 9475.37 |
| -0.0400069 | 4778.68 |
| -0.0627955 | 9877.27 |
| 0.0010535 | -1157.42 |
| -0.007197 | 4065.66 |
| -0.005152 | 3911.91 |
| -0.0478145 | 8254.56 |
| -0.0527731 | 11138.5 |
| 0.0204606 | -9693.43 |
| -0.0227155 | 7836.95 |
| -0.0240912 | 6179.48 |
| 0.0009603 | 7703.16 |
| -0.0240022 | 11230.8 |
| -0.0191314 | 115.66 |
| -0.0189453 | 5204.31 |
| -0.0001103 | 6603.86 |
| -0.0281873 | 2790.07 |
| -0.0525417 | 21108.2 |
| 0.0251252 | -12337.3 |
| -0.0329723 | 11566.3 |
| -0.0101191 | -8143.84 |
| 0.0176757 | -11507.3 |
| -0.021987 | 10154.8 |
| -0.0271103 | 521.92 |
| 0.0083868 | 890.3 |
| -0.00105 | 4404.35 |
| -0.0460264 | 11959.1 |
| -0.0117566 | 7394.57 |
| -0.0268586 | 8128.1 |
| -0.0352612 | 12278.1 |
| -0.0479428 | 9518.08 |
| -0.0051879 | 8399.74 |
| -0.0538711 | 12754 |
| -0.0238828 | 4624.58 |
| -0.0164972 | 5396.56 |
| 0.0103638 | 8057.06 |
| -0.0184783 | 8417.97 |
| -0.0309691 | 4893.75 |
| -0.0175887 | -1027.65 |
| 0.0120195 | -280.3 |
| -0.0137092 | 4848.26 |
| -0.0363053 | 6225.21 |
| -0.0168996 | 1643.51 |
| -0.0343858 | 6928.38 |
| -0.0088123 | 4913.52 |
| -0.0054523 | 7636.85 |
| -0.0598789 | 18248.5 |
| 0.0180197 | -6545.79 |
| -0.0366188 | 8739.54 |
| -0.0349674 | 3364.96 |
| -0.0531723 | 7075.11 |
| -0.0213444 | 9354.08 |
| -0.0065411 | 4783.82 |
| -0.0207852 | 7781.99 |
| 0.0118292 | 4138.35 |
| -0.0236954 | 8275.73 |
| -0.0247432 | 5870.54 |
| -0.0327745 | 4396.19 |
| -0.0155866 | 7629.19 |
| -0.0471108 | 5934.52 |
| -0.0311042 | 11877.8 |
| -0.0134119 | 4943.97 |
| -0.034447 | 4692.99 |
| 0.0082335 | -101.49 |
| -0.0019138 | 808.51 |
| -0.0149995 | 5854.53 |
| -0.033291 | 6909.24 |
| -0.0551121 | 3510.84 |
| -0.0598386 | 11803 |
| -0.0076743 | 2422.8 |
| 0.0031841 | -4836.59 |
| -0.0403357 | 11627.7 |
| -0.0236885 | 10539.4 |
| -0.0447222 | 21340.8 |
| -0.0222249 | 6539.7 |
| 0.0047533 | 5974.9 |
| -0.0268381 | -2702.31 |
| -0.0126129 | 2720.23 |
| -0.0308481 | 16948.7 |
| -0.0158929 | 9261.64 |
| -0.0021679 | 1179.11 |
| -0.0052793 | 18.09 |
| 0.0205562 | -4388.1 |
| -0.0154311 | 6162.57 |
| -0.0204145 | 6310.45 |
| -0.017544 | 11274.3 |
| -0.0187086 | 10115.8 |
| -0.0107079 | -2101.8 |
| -0.0051882 | 5117.64 |
| -0.0243698 | 9706.62 |
| -0.0125956 | 7079.62 |
| -0.0376045 | 13031 |
| -0.0188277 | 3894.07 |
| -0.0260893 | 8189.72 |
| -0.023975 | 3206.73 |
| -0.0155327 | 473.8 |
| -0.0005645 | 1589.2 |
| -0.0161243 | 7306.7 |
| -0.0283685 | 2710.02 |
| -0.0004212 | 5887.98 |
| -0.0122396 | 2855.45 |
| -0.0079486 | 18621.1 |
| -0.0438274 | 7333.71 |
| -0.0689093 | 10816.1 |
| -0.0223773 | 4467.08 |
| -0.0206714 | 33.05 |
| -0.0095789 | 7392.93 |
| -0.0068137 | 6793.79 |
| -0.0338388 | 9654.01 |
| -0.0150156 | 8857.37 |
| -0.0050295 | -1286.92 |
| -0.0326656 | 4994.1 |
| 0.021515 | 5928.01 |
| -0.0177459 | 9373.15 |
| -0.0029376 | 815.28 |
| -0.0206518 | -340.27 |
| 0.0092972 | -5173.27 |
| -0.0432264 | 14155.7 |
| -0.0012667 | -298.45 |
| -0.0206277 | 11845.8 |
| -0.0230132 | 5836.47 |
| 0.0073313 | 5380.95 |
| -0.0244355 | 1503.67 |

Cost-effectiveness acceptability curves

Probability cost-effective

| Willingness to pay threshold | ROBOT SURGERY | OPEN SURGERY |
| --- | --- | --- |
| 0 | 0.152 | 0.848 |
| 10000 | 0.144 | 0.856 |
| 20000 | 0.137 | 0.863 |
| 30000 | 0.137 | 0.863 |
| 40000 | 0.134 | 0.866 |
| 50000 | 0.131 | 0.869 |
| 60000 | 0.129 | 0.871 |
| 70000 | 0.129 | 0.871 |
| 80000 | 0.128 | 0.872 |
| 90000 | 0.127 | 0.873 |
| 120000 | 0.127 | 0.873 |
| 130000 | 0.127 | 0.873 |
| 140000 | 0.128 | 0.872 |
| 150000 | 0.129 | 0.871 |
| 160000 | 0.129 | 0.871 |
| 170000 | 0.129 | 0.871 |

|  | EVPI |
| --- | --- |
| 0 | 612.883 |
| 10000 | 610.869 |
| 20000 | 610.627 |
| 30000 | 610.959 |
| 40000 | 611.826 |
| 50000 | 613.656 |
| 60000 | 615.727 |
| 70000 | 618.539 |
| 80000 | 622.086 |
| 90000 | 626.749 |
| 120000 | 642.359 |
| 130000 | 648.13 |
| 140000 | 654.124 |
| 150000 | 660.172 |
| 160000 | 666.412 |
| 170000 | 672.652 |

Legend supplementary figure 10: Upper figure: the cost-difference between RPD and OPD (Y-axis, euro) and the QALY-difference (X-axis). Lower figure: The probability of cost-effectiveness (Y-axis, rate from 0 to 1) for different willingness-to-pay thresholds (X-axis, euro).

## Supplementary figure 11: Cost-effectiveness of RPD versus OPD in the DIPLOMA-2 randomized trial up to 6 months in high-risk patients only

Cost-effectiveness plane

|  |  |
| --- | --- |
| -0.0018961 | -6526.83 |
| 0.0138583 | -2735.35 |
| -0.0036972 | 2930.6 |
| 0.0204813 | -2813.17 |
| -0.013927 | -1755.06 |
| -0.0019815 | 9298.63 |
| -0.0286509 | 15154.7 |
| -0.0113566 | 8594.48 |
| -0.0161441 | 3008.25 |
| -0.0286066 | 3196.24 |
| -0.0074321 | 4291.26 |
| 0.0001083 | -1133.01 |
| 0.0095518 | 2585.21 |
| -0.0249938 | 6151.51 |
| -0.0011235 | -3349.22 |
| -0.0028047 | -1713.3 |
| 0.0042463 | 7857.46 |
| -0.0177222 | 3162.61 |
| 0.007572 | 126.03 |
| -0.0251559 | 10051.7 |
| 0.0133052 | 2231.18 |
| -0.0185212 | -9521.18 |
| -0.0090521 | 1811.41 |
| -0.0181092 | 7317.33 |
| -0.0036098 | 1645.21 |
| -0.0093301 | 3447.83 |
| 0.0012136 | 243.12 |
| -0.0071384 | -3054.38 |
| -0.0322373 | -1436.25 |
| -0.0223629 | -2130.53 |
| -0.0043341 | -5540.71 |
| -0.0108847 | 2801.62 |
| -0.0389551 | 4894.31 |
| -0.0029986 | -3869.44 |
| -0.0032655 | 2937.17 |
| -0.0040289 | 8930.41 |
| 0.0095958 | -13341.8 |
| 0.0024554 | 6029.52 |
| -0.0075183 | 7680.94 |
| 0.0107411 | 4128.32 |
| -0.016918 | 7288.18 |
| -0.0085476 | -8537.9 |
| -0.0261587 | 7692.47 |
| -0.0075823 | 4907.65 |
| -0.0360673 | 13174.1 |
| -0.0210092 | 10332.5 |
| 0.0004528 | -801.54 |
| -0.0157887 | 12854.5 |
| -0.0244752 | 841.78 |
| 0.0165858 | -3846.72 |
| -0.0373837 | 9628.92 |
| 0.0072229 | -64.16 |
| -0.0275207 | -12.8 |
| -0.0345886 | 12283.8 |
| -0.0263004 | 9491.13 |
| -0.0307955 | 7138.18 |
| -0.0277799 | 6908.24 |
| 0.010947 | -7195.68 |
| -0.0209572 | 4325.91 |
| 0.0064775 | 3512.5 |
| -0.0156507 | 7158.7 |
| -0.0193933 | 9192.67 |
| -0.0184363 | 12402.1 |
| -0.001765 | -5230.57 |
| -0.0181621 | 6893.47 |
| -0.0369899 | 5284.98 |
| -0.0153803 | 2034.42 |
| -0.0202601 | 7052.3 |
| -0.0105623 | 2563.53 |
| -0.0068582 | 5795.15 |
| -0.0079584 | 2168.13 |
| -0.0197318 | 2518.51 |
| 3.06e-05 | 3974.16 |
| 0.0009563 | -488.22 |
| 0.0039746 | -753.32 |
| 0.0022559 | -1168.38 |
| -0.0148711 | 5416.67 |
| 0.001355 | -3474.37 |
| -0.0410189 | 5542.45 |
| 0.0201384 | -13648.4 |
| -0.0121493 | 6364.55 |
| -0.0287859 | 4500.3 |
| 0.0138286 | -4653.69 |
| -0.0199047 | 1349.29 |
| -0.0099842 | 8640.53 |
| -0.0313397 | 3588.93 |
| -0.0127817 | 10534.7 |
| -0.006195 | 6452.36 |
| -0.0009346 | -231.12 |
| -0.0066372 | 8615.15 |
| -0.0083275 | 3995.58 |
| -0.0159549 | -2856.66 |
| 0.0104394 | -1624.4 |
| 0.0117986 | -3377.39 |
| -0.026625 | 1382.04 |
| -0.0324876 | 1297.52 |
| -0.0214665 | -911.03 |
| -0.0164983 | 10449.9 |
| -0.0378034 | 4583.82 |
| 0.0226261 | -229.07 |
| -0.0274949 | 2973.69 |
| 0.0230085 | -4075.03 |
| -0.0195748 | -1914.94 |
| -0.0010071 | 428.58 |
| -0.0249664 | 1910.25 |
| -0.0154157 | 521.44 |
| -0.0077288 | 2445.6 |
| -0.0031776 | -8752.02 |
| -0.012198 | 4442.53 |
| -0.0221784 | 5384.82 |
| -0.0160357 | -1874.94 |
| -0.0161526 | 8626.28 |
| -0.0139696 | 438.91 |
| 0.0051263 | 2204.8 |
| -0.0173159 | -2828.37 |
| 0.0015088 | -3091.52 |
| -0.009458 | 2905.81 |
| 0.0046965 | -1296.07 |
| -0.0105795 | -7340.79 |
| -0.014961 | -2773.58 |
| 0.0009664 | -3748.12 |
| -0.0208617 | 4529.98 |
| 0.0022687 | 2177.95 |
| 0.0134713 | 405.35 |
| -0.0112644 | -4270.54 |
| 0.0060534 | -2623.17 |
| 0.0035988 | 1521.76 |
| 0.0030983 | 1959.97 |
| -0.0277143 | 1918.15 |
| -0.0059272 | 4536.9 |
| -0.0383433 | 8131.89 |
| -0.0129137 | 4452.62 |
| -0.0231046 | 2321.28 |
| -0.0153497 | -599.23 |
| -0.0042055 | 2564.71 |
| 0.0116683 | 154.05 |
| -0.0050322 | 4093.55 |
| -0.0145456 | 2806.48 |
| 0.0075819 | 4602.87 |
| -0.0289201 | 6961.57 |
| 0.003055 | -761.33 |
| -0.0154803 | -1650.84 |
| -0.0209876 | 5483.29 |
| -0.0036443 | -6357.51 |
| -0.0038962 | -1099.61 |
| -0.008976 | 5270.62 |
| 0.003752 | -76.89 |
| -0.0331217 | 8267.23 |
| -0.0090899 | 1252.48 |
| -0.0364262 | 3546.35 |
| -0.0194174 | 3158.1 |
| -0.0024159 | 2361.09 |
| -0.0255311 | 183.03 |
| -0.0112859 | -381.99 |
| -0.0068481 | -1030.91 |
| 0.0029973 | 1084.34 |
| -0.0141842 | 6797.02 |
| -0.0063808 | -755.26 |
| -0.0202611 | -5011.31 |
| -0.0158386 | 4587.88 |
| -4.3e-05 | 5757.51 |
| -0.0203095 | 3641.77 |
| -0.0023071 | 2798.36 |
| -0.0069557 | 5143 |
| -0.0470612 | 7415.91 |
| -0.0063792 | 4802.78 |
| -0.0026597 | 5146.81 |
| 0.0043297 | 5043.18 |
| -0.003034 | -1074.4 |
| 0.018264 | 1909.63 |
| -0.0017696 | 209.36 |
| -0.0023774 | -1101.39 |
| 0.0062981 | 1441.47 |
| -0.0119007 | 1914.17 |
| -0.005126 | 2229.63 |
| -0.0052553 | -1649.95 |
| -0.0163728 | 7437.45 |
| -0.0008474 | -4782.11 |
| -0.0249814 | 4138.71 |
| -0.0075872 | 3072.59 |
| -0.0223084 | 8691.91 |
| -0.0178276 | 4608.75 |
| 0.0147816 | -389.03 |
| -0.0125486 | 167.46 |
| -0.0037285 | 6429.8 |
| 0.0027024 | -7767.86 |
| -0.0195104 | 4941.85 |
| -0.0250359 | 4639.01 |
| -0.0185735 | -3889.95 |
| -0.0171862 | 9947.34 |
| 0.0136362 | -5095.65 |
| -0.0215021 | 4177.2 |
| -0.0131601 | 5808.32 |
| -0.005173 | 2358.17 |
| 0.0087805 | -1149.77 |
| 0.0073169 | 1090.08 |
| -0.0111221 | 14278.9 |
| -0.0192301 | 8736.27 |
| -0.031939 | 2611.95 |
| -0.0279352 | 11361.6 |

Cost-effectiveness acceptability curves

Probability cost-effective

| Willingness to pay threshold | ROBOT SURGERY | OPEN SURGERY |
| --- | --- | --- |
| 0 | 0.318 | 0.682 |
| 10000 | 0.312 | 0.688 |
| 20000 | 0.311 | 0.689 |
| 30000 | 0.31 | 0.69 |
| 40000 | 0.304 | 0.696 |
| 50000 | 0.3 | 0.7 |
| 60000 | 0.302 | 0.698 |
| 70000 | 0.299 | 0.701 |
| 80000 | 0.302 | 0.698 |
| 90000 | 0.3 | 0.7 |
| 120000 | 0.297 | 0.703 |
| 130000 | 0.294 | 0.706 |
| 140000 | 0.288 | 0.712 |
| 150000 | 0.291 | 0.709 |
| 160000 | 0.29 | 0.71 |
| 170000 | 0.289 | 0.711 |

|  | EVPI |
| --- | --- |
| 0 | 1186.98 |
| 10000 | 1188.98 |
| 20000 | 1191.78 |
| 30000 | 1195.27 |
| 40000 | 1199.71 |
| 50000 | 1206.02 |
| 60000 | 1212.98 |
| 70000 | 1220.62 |
| 80000 | 1229.17 |
| 90000 | 1238.56 |
| 120000 | 1271.83 |
| 130000 | 1284.36 |
| 140000 | 1297.89 |
| 150000 | 1312.04 |
| 160000 | 1326.74 |
| 170000 | 1341.91 |

Legend supplementary figure 11: Upper figure: the cost-difference between RPD and OPD (Y-axis, euro) and the QALY-difference (X-axis). Lower figure: The probability of cost-effectiveness (Y-axis, rate from 0 to 1) for different willingness-to-pay thresholds (X-axis, euro).

## Supplementary figure 12: Cost-effectiveness of RPD versus OPD in the DIPLOMA-2 randomized trial up to 6 months in low-risk patients only

Cost-effectiveness plane

|  |  |
| --- | --- |
| -0.0615566 | 7426.68 |
| -0.0034865 | -688.11 |
| -0.0346992 | 7145.69 |
| -0.0332796 | 1584.05 |
| -0.0160409 | 1558.34 |
| -0.0323139 | 5554.91 |
| -0.0270863 | 7030.55 |
| -0.0242824 | -8233.13 |
| -0.0305884 | 7643.57 |
| -0.0692432 | 12558.3 |
| -0.0399682 | 5383.38 |
| -0.035383 | 383.9 |
| -0.0497836 | 8953.75 |
| -0.0300752 | 8426.61 |
| -0.0433493 | 3099.53 |
| -0.0311449 | 5331.97 |
| -0.0760465 | 10496.6 |
| -0.0443117 | 9673.74 |
| -0.0703028 | 223.04 |
| -0.0145954 | -734.31 |
| -0.0478411 | 7590.93 |
| -0.0254853 | 7104.74 |
| -0.0360657 | 7492.65 |
| -0.0541744 | 12424.9 |
| -0.0397292 | 11072.9 |
| -0.0448474 | 5207.29 |
| -0.0350315 | 1148.18 |
| -0.0554903 | 15501.1 |
| -0.0261317 | -5226.73 |
| -0.0483392 | -6109.01 |
| -0.0088053 | 3385.53 |
| -0.0370682 | 4587.31 |
| -0.0272752 | -13389.8 |
| -0.052068 | 8841.83 |
| -0.0285463 | -4773.99 |
| -0.0514681 | 3980.21 |
| -0.0634069 | 9369.2 |
| -0.0587547 | 13656 |
| -0.0234546 | -901.66 |
| -0.0509971 | 12093.8 |
| -0.0316511 | 658.78 |
| -0.047576 | 6992.52 |
| -0.0302662 | -7341.29 |
| -0.0259024 | -3220.64 |
| -0.0140322 | 11155.7 |
| -0.0320514 | 4957.22 |
| -0.0361424 | 3620.78 |
| -0.0483924 | 7395.71 |
| -0.0341565 | 648.13 |
| -0.0378419 | 4409.55 |
| -0.0349806 | 5543.43 |
| -0.0238544 | 2465.78 |
| -0.0482602 | 6866.06 |
| -0.0320666 | 4320.76 |
| -0.0332788 | 7282.03 |
| -0.025001 | -1684.06 |
| -0.0278779 | -2723.57 |
| -0.0329715 | -4896.69 |
| -0.023918 | 3117.64 |
| -0.0195935 | 337.25 |
| -0.0368829 | -1167.87 |
| 0.0062532 | -19803.4 |
| -0.0142701 | -25134.5 |
| -0.057187 | -2069.76 |
| -0.0582352 | 6776.08 |
| -0.0378404 | 5918.91 |
| -0.0163212 | -5548.39 |
| -0.0432251 | 14566.1 |
| -0.0496327 | 1074.05 |
| -0.0503828 | 12037 |
| -0.0269316 | -4865.18 |
| -0.0319747 | 17487.3 |
| -0.0514888 | 15432.8 |
| -0.0363158 | 4027.66 |
| -0.0233099 | -6458.78 |
| -0.0148229 | -7162.32 |
| -0.0282851 | -3826.58 |
| -0.0343125 | 9818.01 |
| -0.0141485 | -5933.46 |
| -0.0430679 | -6109.99 |
| -0.018331 | -5012.9 |
| -0.0217316 | -6454.04 |
| 2.03e-05 | -770.03 |
| 0.0020125 | -10174.9 |
| -0.0158481 | -944.36 |
| -0.0102061 | -15319.1 |
| -0.0495774 | 5852.2 |
| -0.0344878 | -7533.58 |
| -0.0015914 | 4609.88 |
| -0.0234218 | 4236.41 |
| -0.0300024 | 5924.8 |
| -0.0233082 | 316.08 |
| -0.0202425 | -9082.39 |
| -0.0191704 | 481.55 |
| -0.0371058 | 6982.67 |
| -0.0542204 | 4645.48 |
| -0.010393 | -6585.73 |
| -0.002784 | -3566.6 |
| 0.0056589 | 1631.51 |
| -0.037246 | -3378.11 |
| -0.0332171 | 10525.8 |
| -0.0570281 | 6661.97 |
| -0.0578262 | 7577.15 |
| -0.0444554 | 9354.76 |
| -0.0289963 | -3874.84 |
| -0.0129144 | -6862.2 |
| -0.0564961 | 4388.58 |
| -0.0390611 | 7223.46 |
| -0.0256642 | 5555.79 |
| -0.0483136 | 11389.7 |
| -0.0144148 | -4798.73 |
| -0.0185255 | 3630.03 |
| -0.0599224 | -6271.97 |
| -0.0077605 | 8941.33 |
| -0.0153479 | 10255.3 |
| -0.0579646 | 1536.9 |
| -0.061523 | 15626.3 |
| -0.060084 | 8408.11 |
| -0.0444492 | 6983.53 |
| -0.0276841 | 6557.93 |
| -0.0453951 | 7599.03 |
| -0.0476608 | 4864 |
| 0.0066937 | -3909.26 |
| -0.037734 | 12001.6 |
| -0.0377101 | 1995.42 |
| -0.0277533 | -4593.81 |
| -0.0308943 | -9033.74 |
| -0.042677 | 12950.2 |
| -0.0329478 | -833.94 |
| -0.0280053 | -1321.02 |
| 0.006841 | -12112.1 |
| -0.0693634 | 18173.7 |
| -0.0067914 | 2844.88 |
| -0.0546585 | 5477.57 |
| 0.0024243 | -22426.8 |
| -0.0493082 | 15123.4 |
| -0.0276904 | -3379.84 |
| -0.0204692 | 11547.8 |
| -0.0405301 | 5014.78 |
| -0.0108018 | -5149.87 |
| -0.029042 | 5745.77 |
| -0.0340176 | 279.83 |
| -0.0077933 | -10663.1 |
| -0.0047043 | 645.7 |
| -0.0633319 | 7864.16 |
| -0.0301234 | -2464.86 |
| -0.0306262 | 8326.27 |
| -0.050684 | 5937.41 |
| -0.0519647 | 7333.91 |
| -0.0521226 | 9656.15 |
| -0.0435781 | 14440.3 |
| -0.0282732 | 664.14 |
| -0.0484304 | 8602.53 |
| -0.0523293 | 11825.1 |
| -0.0054452 | 12.25 |
| -0.0347847 | 4733.87 |
| -0.0289498 | 10804.2 |
| -0.0576008 | -7005.88 |
| -0.0316961 | -13783.5 |
| -0.0010779 | 246.67 |
| -0.0663347 | 15880.2 |
| -0.0286267 | 4654.45 |
| -0.0363096 | 3152.36 |
| -0.0036793 | -14202.8 |
| -0.0271382 | 4969.12 |
| 0.007122 | -6313.93 |
| -0.0250871 | 6422.5 |
| -0.0292367 | 3636.28 |
| -0.0179728 | 949.2 |
| -0.0354483 | 8610.26 |
| -0.00953 | -267.73 |
| -0.0746729 | 10831.2 |
| -0.016091 | -629.69 |
| -0.029509 | -8150.17 |
| -0.0295703 | 2632.28 |
| -0.0303887 | 9121.15 |
| -0.0471298 | -3075.72 |
| -0.0336551 | 5094.64 |
| -0.0161883 | -4128.54 |
| -0.0431304 | -7095.85 |
| -0.0320194 | 9215.13 |
| -0.010967 | 1963.14 |
| -0.0112055 | 9840.02 |
| -0.0839397 | 13720.5 |
| -0.0505824 | 8156.48 |
| -0.069476 | 11794.8 |
| -0.028669 | 13427.5 |
| -0.0553299 | 13328.6 |
| -0.0389967 | 15404.4 |
| -0.0335218 | -3021.21 |
| -0.0141976 | -2882.31 |
| -0.0361721 | 3873.28 |
| -0.0462192 | 2752.03 |
| -0.0037104 | -14867.3 |
| -0.022922 | 1463.35 |
| -0.0490461 | 874.48 |
| -0.0592424 | 9469.98 |
| -0.0600456 | 10789 |
| -0.0623323 | 12681.8 |
| -0.0643857 | 2206.94 |

Cost-effectiveness acceptability curves

Probability cost-effective

| Willingness to pay threshold | ROBOT SURGERY | OPEN SURGERY |
| --- | --- | --- |
| 0 | 0.294 | 0.706 |
| 10000 | 0.287 | 0.713 |
| 20000 | 0.274 | 0.726 |
| 30000 | 0.266 | 0.734 |
| 40000 | 0.253 | 0.747 |
| 50000 | 0.243 | 0.757 |
| 60000 | 0.235 | 0.765 |
| 70000 | 0.228 | 0.772 |
| 80000 | 0.223 | 0.777 |
| 90000 | 0.217 | 0.783 |
| 120000 | 0.194 | 0.806 |
| 130000 | 0.185 | 0.815 |
| 140000 | 0.176 | 0.824 |
| 150000 | 0.169 | 0.831 |
| 160000 | 0.164 | 0.836 |
| 170000 | 0.159 | 0.841 |

|  | EVPI |
| --- | --- |
| 0 | 1701.29 |
| 10000 | 1636.91 |
| 20000 | 1576.01 |
| 30000 | 1518.66 |
| 40000 | 1464.72 |
| 50000 | 1415 |
| 60000 | 1367.38 |
| 70000 | 1321.86 |
| 80000 | 1278.24 |
| 90000 | 1237.53 |
| 120000 | 1130.75 |
| 130000 | 1099.33 |
| 140000 | 1070.66 |
| 150000 | 1044.72 |
| 160000 | 1020.41 |
| 170000 | 997.363 |

Legend supplementary figure 12: Upper figure: the cost-difference between RPD and OPD (Y-axis, euro) and the QALY-difference (X-axis). Lower figure: The probability of cost-effectiveness (Y-axis, rate from 0 to 1) for different willingness-to-pay thresholds (X-axis, euro).

## References

1.	de Graaf, N., et al., *Minimally Invasive versus Open Pancreatoduodenectomy for Resectable Neoplasms.* NEJM Evid, 2025. **4**(12): p. EVIDoa2500045.

2.	Horvath, B., et al., *The Evolution, Current Value, and Future of the American Society of Anesthesiologists Physical Status Classification System.* Anesthesiology, 2021. **135**(5): p. 904–919.

3.	Fiona Campbell CA, D.F., Feakins R. , *Dataset for histopathological reporting of carcinomas of the pancreas, ampulla of Vater and common bile duct.* The Royal College of Pathologists, 2019.

4.	Clavien, P.A., et al., *The Clavien-Dindo classification of surgical complications: five-year experience.* Ann Surg, 2009. **250**(2): p. 187–96.

5.	Bassi, C., et al., *The 2016 update of the International Study Group (ISGPS) definition and grading of postoperative pancreatic fistula: 11 Years After.* Surgery, 2017. **161**(3): p. 584–591.

6.	Wente, M.N., et al., *Postpancreatectomy hemorrhage (PPH): an International Study Group of Pancreatic Surgery (ISGPS) definition.* Surgery, 2007. **142**(1): p. 20–5.

7.	Wente, M.N., et al., *Delayed gastric emptying (DGE) after pancreatic surgery: a suggested definition by the International Study Group of Pancreatic Surgery (ISGPS).* Surgery, 2007. **142**(5): p. 761–8.

8.	Koch, M., et al., *Bile leakage after hepatobiliary and pancreatic surgery: A definition and grading of severity by the International Study Group of Liver Surgery.* Surgery, 2011.

9.	Garner, J.S., *CDC guideline for prevention of surgical wound infections, 1985. Supersedes guideline for prevention of surgical wound infections published in 1982. (Originally published in November 1985). Revised.* Infect Control, 1986. **7**(3): p. 193–200.

10.	de Rooij, T., et al., *Minimally Invasive Versus Open Distal Pancreatectomy (LEOPARD): A Multicenter Patient-blinded Randomized Controlled Trial.* Ann Surg, 2019. **269**(1): p. 2–9.

11.	Menso, J.E., et al., *Cost-analysis of Implementing robot-assisted Versus Open Pancreatoduodenectomy.* Ann Surg, 2025.

12.	Patel, S., et al., *Understanding the Costs of Surgery: A Bottom-Up Cost Analysis of Both a Hybrid Operating Room and Conventional Operating Room.* Int J Health Policy Manag, 2022. **11**(3): p. 299–307.

13.	Herdman, M., et al., *Development and preliminary testing of the new five-level version of EQ-5D (EQ-5D-5L).* Qual Life Res, 2011. **20**(10): p. 1727–36.

14.	Fayers, P., et al., *Quality of life research within the EORTC-the EORTC QLQ-C30. European Organisation for Research and Treatment of Cancer.* Eur J Cancer, 2002. **38 Suppl 4**: p. S125–33.

15.	Fitzsimmons, D., et al., *Development of a disease specific quality of life (QoL) questionnaire module to supplement the EORTC core cancer QoL questionnaire, the QLQ-C30 in patients with pancreatic cancer. EORTC Study Group on Quality of Life.* Eur J Cancer, 1999. **35**(6): p. 939–41.

16.	Bassi, C., et al., *The 2016 update of the International Study Group (ISGPS) definition and grading of postoperative pancreatic fistula: 11 Years After.* Surgery, 2017. **161**(3): p. 584–591.

17.	Suurmeijer, J.A., et al., *Nationwide validation of the ISGPS risk classification for postoperative pancreatic fistula after pancreatoduodenectomy: "Less is more".* Surgery, 2023. **173**(5): p. 1248–1253.

18.	White, I.R. and S.G. Thompson, *Adjusting for partially missing baseline measurements in randomized trials.* Stat Med, 2005. **24**(7): p. 993–1007.

19.	Faria, R., et al., *A guide to handling missing data in cost-effectiveness analysis conducted within randomised controlled trials.* Pharmacoeconomics, 2014. **32**(12): p. 1157–70.

20.	Senn, S.J., *Covariate imbalance and random allocation in clinical trials.* Stat Med, 1989. **8**(4): p. 467–75.

21.	Manca, A., N. Hawkins, and M.J. Sculpher, *Estimating mean QALYs in trial-based cost-effectiveness analysis: the importance of controlling for baseline utility.* Health Econ, 2005. **14**(5): p. 487–96.

22.	Kahan, B.C. and T.P. Morris, *Improper analysis of trials randomised using stratified blocks or minimisation.* Stat Med, 2012. **31**(4): p. 328–40.

23.	Amdal, C.D., et al., *SISAQOL-IMI consensus-based guidelines to design, analyse, interpret, and present patient-reported outcomes in cancer clinical trials.* Lancet Oncol, 2025. **26**(12): p. e683–e693.

24.	Leona Hakkaart-van Roijen, S.P., Tim Kanters in opdracht van Zorginstituut Nederland, *Kostenhandleiding voor economische evaluaties in de gezondheidszorg: Methodologie en Referentieprijzen. Herziene versie 2024*. 2024, Zorginstituut Nederland.
