# Supplementary material for: Cost-effectiveness Analysis of Robotic Versus Open Pancreatoduodenectomy (DIPLOMA-2): A Multicenter International Randomized Trial
Source: Ann Surg. 2026 Aug 14;284(5):920–7. doi: 10.1097/SLA.0000000000007188 (PMC13652126; doi:10.1097/SLA.0000000000007188)
Supplement: Supplementary file 2 [file sla-284-0920-s002.docx]

# Table 1: Baseline characteristics of patients undergoing RPD versus OPD in the DIPLOMA-2 randomized trial

| **Baseline characteristics** | **RPD**  **(n=170)** | **OPD**  **(n=98)** |
| --- | --- | --- |
| Age (years) | 72 (63–76) | 69 (59–75) |
| Female | 68 (40.0%) | 42 (42.9%) |
| BMI (kg/m^2^) | 24.8 (22.1–27.6) | 24.8 (23.0–27.6) |
| ASA score ≥3 | 55 (32.4%) | 33 (33.7%) |
| Comorbidities | 126 (74.1%) | 75 (76.5%) |
| Preoperative pancreatitis | 1 (0.6%) | 2 (2.0%) |
| Preoperative diabetes | 32 (18.8%) | 21 (21.4%) |
| Cardiac disease | 38 (22.4%) | 22 (22.4%) |
| Pulmonary disease | 18 (10.6%) | 10 (10.2%) |
| Previous abdominal surgery  *Missing* | 59 (35.1%)  *2* | 29 (29.6%)  *0* |
| Indication PDAC  *Missing* | 49 (28.8%)  *0* | 22 (22.9%)  *2* |
| High fistula risk* | 110 (64.7%) | 60 (61.2%) |

Legend table 1: Values are presented in medians with interquartile ranges and frequencies with percentages. RPD, robot-assisted pancreatoduodenectomy; OPD, open pancreatoduodenectomy; ASA, American Society of Anesthesiologists; PDAC, pancreatic ductal adenocarcinoma. *Based on MPD <3 mm and/or BMI >25 kg/m^2^.
