# Supplementary material for: Cost-effectiveness Analysis of Robotic Versus Open Pancreatoduodenectomy (DIPLOMA-2): A Multicenter International Randomized Trial
Source: Ann Surg. 2026 Aug 14;284(5):920–7. doi: 10.1097/SLA.0000000000007188 (PMC13652126; doi:10.1097/SLA.0000000000007188)
Supplement: Supplementary file 3 [file sla-284-0920-s003.docx]

# Table 3: Hospital costs for patients undergoing RPD versus OPD in the DIPLOMA-2 randomized trial up to 6 months

| **Costs per patient (euro)** | **RPD**  **(n=170)** | **OPD**  **(n=98)** | **P-value** | **Mean difference (euro)** |
| --- | --- | --- | --- | --- |
| **Intraoperative costs** | **11,906 (11,633–12,193)** | **6,451 (6,122–6,712)** | **<0.001** | **5,491 (5,086 to 5,903)** |
| Fixed | 6,548 (6,465–6,627) | 2,212 (2,109–2,309) | **<0.001** | 4,337 (4,211 to 4,465) |
| Additional | 5,357 (5,089–5,646) | 4,204 (3,936–4,476) | **<0.001** | 1,154 (771 to 1,545) |
| **Postoperative costs** | **19,051 (15,714–22,695)** | **21,856 (15,628–29,794)** | **0.518** | **-2,806 (-10,488 to 4,877)** |
| Admission | 16,346 (13,284–20,000) | 19,189 (13,627–26,165) | 0.472 | -2,844 (-10,217 to 3,660) |
| Unplanned hospital visits | 89 (65–117) | 64 (44–86) | 0.178 | 25 (-9 to 61) |
| Endoscopic interventions | 448 (320–591) | 420 (248–616) | 0.821 | 29 (-211 to 258) |
| Radiologic interventions | 1,089 (780–1,437) | 1,323 (891–1,823) | 0.463 | -235 (-822 to 330) |
| Surgical reinterventions | 802 (486–1,152) | 482 (235–787) | 0.199 | 320 (-176 to 807) |
| Other | 277 (169–402) | 378 (146–674) | 0.560 | -101 (-390 to 188) |
| **Total costs*** | **30,956 (27,557–34,655)** | **28,271 (21,999–36,257)** | **0.538** | **2,685 (-5,072 to 10,442)** |
| Excl robot purchase costs | 28,806 (25,407–32,505) | 28,271 (21,999–36,257) | 0.898 | 535 (-7,222 to 8,292) |
| In patients with non-PDAC | 32,338 (28,146–37,042) | 27,630 (21,793–34,645) | 0.317 | 4,709 (-3,796 to 13,214) |
| In patients with PDAC | 27,544 (23,055–33,440) | 31,852 (16,559–55,051) | 0.733 | -4,308 (-28,753 to 13,063) |
| In high-risk patients# | 31,493 (26,959–36,627) | 29,224 (21,973–38,764) | 0.686 | 2,270 (-8,616 to 11,486) |
| In low-risk patients^ | 29,972 (24,962–36,251) | 26,768 (18,061–41,339) | 0.716 | 3,204 (-10,217–16,625) |

Legend table 3: Costs are presented in means with mean differences and bias-corrected and accelerated 95% confidence intervals (95% BCa CIs). *Intra- and postoperative costs. #Based on MPD ≥3 mm and BMI ≤25 kg/m^2^. ^Based on MPD <3 mm and BMI >25 kg/m^2^.
