# Supplementary material for: Cost-effectiveness Analysis of Robotic Versus Open Pancreatoduodenectomy (DIPLOMA-2): A Multicenter International Randomized Trial
Source: Ann Surg. 2026 Aug 14;284(5):920–7. doi: 10.1097/SLA.0000000000007188 (PMC13652126; doi:10.1097/SLA.0000000000007188)
Supplement: Supplementary file 4 [file sla-284-0920-s004.docx]

# Figure 3: Cost-effectiveness in all patients undergoing RPD vs OPD in the DIPLOMA-2 randomized trial up to 6 months

Cost-effectiveness plane

|  |  |
| --- | --- |
| -0.0120685 | 2647.83 |
| -0.0135741 | -686.96 |
| -0.0026795 | -6372.75 |
| -0.0190761 | 2822.25 |
| -0.0300774 | 7032.41 |
| -0.0238388 | 4433.33 |
| 0.0021483 | -2103.23 |
| -0.0015928 | -3879.83 |
| -0.0210566 | 3517.18 |
| -0.0140941 | -7428.52 |
| -0.0335176 | 4666.29 |
| -0.0143776 | -1833.43 |
| -0.032216 | 11125 |
| -0.0035279 | -2209.04 |
| -0.0035948 | 4085.86 |
| -0.027707 | 5268.94 |
| -0.0246058 | -6548.21 |
| -0.0094098 | -432.76 |
| -0.0128338 | 380.44 |
| -0.0287768 | 3836.4 |
| -0.0363919 | 5438.9 |
| -0.0451181 | 6306.27 |
| -0.0237133 | 4196.19 |
| -0.0252505 | 6354.27 |
| -0.0127291 | 4016.38 |
| -0.0260539 | -3186.1 |
| -0.0226132 | 5030.69 |
| -0.0240842 | 5641.24 |
| -0.023619 | 5774.39 |
| -0.0239894 | 3059.73 |
| -0.0305071 | 7651.19 |
| -0.0290789 | 3434.68 |
| -0.0083802 | -514.9 |
| -0.0185677 | 234.07 |
| -0.0060976 | 2969.82 |
| -0.0352018 | 4752.98 |
| -0.0143314 | 5421 |
| -0.0194141 | 5607.73 |
| -0.0100918 | 2607.23 |
| 0.000821 | -4440.08 |
| -0.0134047 | 6035.04 |
| -0.0274466 | 9110.96 |
| -0.0284378 | 8091.48 |
| -0.012136 | -5094.36 |
| -0.0232241 | -1046.36 |
| -0.0322264 | 8819.84 |
| -0.0122812 | 499 |
| -0.0355572 | 3399.1 |
| -0.0199721 | 3676.48 |
| -0.0386297 | 9015.49 |
| -0.0114401 | 2658.62 |
| -0.0099448 | -6467.95 |
| -0.0071109 | 3196 |
| -0.0360425 | 6728.68 |
| -0.0185614 | 5263.77 |
| -0.0107186 | 4383.21 |
| -0.0214469 | 2729.43 |
| -0.0014876 | -606.53 |
| -0.0187631 | 4302.38 |
| -0.0204919 | 26.63 |
| -0.0262459 | 63.67 |
| -0.0286484 | 7058.02 |
| -0.0210572 | 301.75 |
| -0.0136702 | 3985.44 |
| 0.004104 | -12549.3 |
| -0.0037126 | -381 |
| -0.0177483 | 3399.94 |
| -0.0318133 | -2279.82 |
| 0.0097133 | -1442.94 |
| -0.0282337 | 6700.19 |
| -0.0082298 | 3739.79 |
| -0.017947 | 1548.43 |
| -0.0283463 | 3484.2 |
| -0.0009699 | -2071.55 |
| -0.001474 | -1030.54 |
| -0.0353553 | 10361.6 |
| -0.0064757 | 4271.28 |
| -0.0122629 | 4223.75 |
| -0.0400481 | 10877.2 |
| -0.0166691 | -3565.68 |
| -0.0188926 | 3536.51 |
| -0.0183308 | -1486.04 |
| -0.0252253 | -2510.8 |
| -0.0200702 | 5178.34 |
| -0.0163229 | 3804.53 |
| -0.0300534 | -6703.96 |
| -0.0284342 | 3737.54 |
| -0.0393752 | 9173.52 |
| -0.0362975 | 8918.76 |
| -0.0143977 | 1459.16 |
| -0.0357666 | 6070.41 |
| -0.0075245 | -2540.57 |
| -0.0374615 | 4254.96 |
| 0.0121643 | 1342.7 |
| -0.0205984 | 6060.09 |
| -0.0285294 | 3080.02 |
| -0.0040068 | -9244.83 |
| -0.0176676 | -206.18 |
| -0.0229932 | 2186.11 |
| -0.0243216 | 3666.06 |
| -0.020416 | 4724.5 |
| -0.0419553 | 8096.46 |
| -0.0460741 | 4882.46 |
| -0.034238 | 2515.46 |
| -0.0419127 | 6458.65 |
| -0.0198749 | 8420.84 |
| -0.0252219 | 6790.83 |
| -0.0188602 | 7803.81 |
| -0.0115577 | 2074.35 |
| -0.0398622 | 1675 |
| -0.0144186 | 7359.47 |
| -0.0230114 | 1233.67 |
| -0.0310575 | -1110.8 |
| -0.0110825 | 1892.07 |
| -0.0008358 | -8786.2 |
| -0.036355 | 4626.2 |
| -0.0034908 | 1648.86 |
| -0.0400812 | 8734.93 |
| -0.0194364 | 2160.5 |
| -0.0056596 | 3857.28 |
| -0.0390035 | 995.38 |
| -0.0155657 | 8481.18 |
| -0.0278275 | 8900.86 |
| -0.031234 | 3434.6 |
| -0.0038832 | -6770.24 |
| -0.0149681 | -3882.85 |
| -0.0082958 | -1340.38 |
| -0.0278474 | 6317.07 |
| -0.023485 | -67.25 |
| -0.0080228 | -2734.07 |
| -0.0198761 | 5809.89 |
| -0.0068064 | 4397.04 |
| -0.0227441 | 4502.18 |
| -0.0210174 | 1449.31 |
| -0.0116081 | -3297.14 |
| -0.009843 | 4263.76 |
| -0.0323057 | 2463.18 |
| -0.0181894 | -6078.43 |
| -0.0130884 | -2235.42 |
| -0.0114035 | 4961.65 |
| -0.0216678 | 5397.18 |
| -0.0471782 | 10120.9 |
| 0.0117714 | -10615.5 |
| -0.0179816 | 4080.35 |
| -0.0079005 | 2740.37 |
| -0.0196651 | -1610.6 |
| -0.024766 | 856.49 |
| -0.0196559 | 9741.2 |
| -0.0285456 | 3355.29 |
| -0.02993 | 3510.9 |
| 0.0033774 | -3988.6 |
| -0.0276615 | -1518.32 |
| -0.0331448 | 3907.78 |
| -0.018727 | 3123.56 |
| -0.0404246 | 7040.79 |
| -0.031344 | 5276.04 |
| -0.0012819 | -993.57 |
| -0.0110271 | 1608 |
| -0.0149189 | 1977.72 |
| -0.0289134 | 4830.41 |
| -0.0244997 | -10476.4 |
| -0.0152647 | 5246.58 |
| -0.0084054 | -1129.69 |
| -0.0214724 | -4656.65 |
| -0.0101051 | 4959.32 |
| -0.024783 | 2561.24 |
| -0.0195978 | 9049.29 |
| -0.034664 | 5019.35 |
| -0.0302313 | 9379.57 |
| -0.0294864 | 2753.04 |
| -0.0260711 | -570.25 |
| -0.0135247 | -1321.81 |
| -0.0336489 | 3462.92 |
| -0.0164008 | -412.51 |
| -0.0197077 | 3809.46 |
| -0.0302311 | 6526.91 |
| -0.0063994 | 5964.91 |
| -0.0104979 | 3953.35 |
| 0.0076616 | -2684.42 |
| -0.0158807 | 5975.06 |
| -0.0214111 | -1554.88 |
| -0.0344369 | 5173.76 |
| -0.0327038 | 4593.03 |
| -0.025384 | -1295.12 |
| -0.0152831 | 1236.2 |
| -0.0298304 | 6120.6 |
| -0.0158526 | 6821.67 |
| -0.0115314 | -3813.06 |
| -0.0009292 | -1861.82 |
| -0.0123923 | -5396.87 |
| -0.0198166 | -472.34 |
| -0.0219209 | 3.21 |
| -0.0142849 | 5900.53 |
| -0.0036859 | 912.35 |
| -0.0239616 | 97.54 |
| -0.0167328 | 2301.38 |
| -0.0211616 | 5146.95 |
| -0.031856 | 4521.01 |
| -0.0056459 | -3773.84 |
| -0.0317606 | 11732.9 |

Cost-effectiveness acceptability curves

Probability cost-effective

| Willingness to pay threshold | ROBOT SURGERY | OPEN SURGERY |
| --- | --- | --- |
| 0 | 0.267 | 0.733 |
| 10000 | 0.254 | 0.746 |
| 20000 | 0.246 | 0.754 |
| 30000 | 0.23 | 0.77 |
| 40000 | 0.217 | 0.783 |
| 50000 | 0.206 | 0.794 |
| 60000 | 0.193 | 0.807 |
| 70000 | 0.188 | 0.812 |
| 80000 | 0.181 | 0.819 |
| 90000 | 0.172 | 0.828 |
| 120000 | 0.163 | 0.837 |
| 130000 | 0.158 | 0.842 |
| 140000 | 0.153 | 0.847 |
| 150000 | 0.148 | 0.852 |
| 160000 | 0.145 | 0.855 |
| 170000 | 0.141 | 0.859 |

Legend figure 3: Upper figure: the cost-difference between RPD and OPD (Y-axis, euro) and the QALY-difference (X-axis). Lower figure: The probability of cost-effectiveness (Y-axis, rate from 0 to 1) for different willingness-to-pay thresholds (X-axis, euro). The cost-effectiveness acceptability curve (CEAC) for OPD represents the cost-effectiveness acceptability frontier (CEAF).
